# Mechanical Power as a Predictor of Outcomes During Mechanical Ventilation in Coronavirus Disease 2019 (COVID-19): An Updated Systematic Review

**DOI:** 10.3390/jcm15166476

**Published:** 2026-08-21

**Authors:** Camila Vantini Capasso Palamim, Tais Mendes Camargo, Fernando Augusto Lima Marson

**Affiliations:** 1 Laboratory of Molecular Biology and Genetics, Universidade São Francisco (USF), Bragança Paulista 12916-900, SP, Brazil; cvcpalamim@gmail.com (C.V.C.P.); taismendescamargo@gmail.com (T.M.C.); 2 Laboratory of Clinical and Molecular Microbiology, Universidade São Francisco (USF), Bragança Paulista 12916-900, SP, Brazil; 3 LunGuardian Research Group—Epidemiology of Respiratory and Infectious Diseases, Universidade São Francisco (USF), Bragança Paulista 12916-900, SP, Brazil

**Keywords:** critical care outcomes, lung-protective strategies, respiratory failure, respiratory mechanics, severe acute respiratory syndrome coronavirus 2, ventilator-induced lung injury

## Abstract

**Background/Objectives**: Mechanical power (MP) quantifies the energy delivered to the respiratory system during ventilation and serves as a promising marker for ventilator-induced lung injury (VILI). According to its original definition by Gattinoni, MP reflects the energy transferred from the ventilator to the respiratory system under conditions of deep sedation, passive breathing, neuromuscular blockade, and volume-controlled ventilation. Its role in coronavirus disease 2019 (COVID-19)-associated acute respiratory distress syndrome (ARDS) remains under investigation. This systematic review aimed to synthesize the available evidence on the association between MP and VILI, complications related to mechanical ventilation (MV), and mortality in adult patients with COVID-19 undergoing invasive mechanical ventilation (IMV). **Methods**: A systematic review was conducted using PubMed-MEDLINE (Medical Literature Analysis and Retrieval System Online) for studies published in recent years, focusing on adult COVID-19 patients undergoing IMV. Inclusion criteria centered on studies reporting MP and its association with VILI, complications, or mortality. Ten studies met eligibility criteria after screening 356 retrieved articles. **Results**: Most included studies were retrospective and observational, encompassing critically ill COVID-19 patients. Elevated MP was correlated with more severe outcomes, including increased 28-day mortality, prolonged MV, and weaning failure. Franck et al. demonstrated strong correlations between MP and driving pressure, elastance, and positive end-expiratory pressure, emphasizing the importance of calculation methods. González-Castro et al. identified a threshold of 17 J/min, above which mortality risk increased. Stalla et al. highlighted that dynamic MP reductions during prone positioning were associated with survival. Registry-based analyses confirmed that both magnitude and cumulative exposure above 18 J/min increased intensive care unit mortality. Novel indices combining MP with oxygenation parameters improved prognostic accuracy. While absolute MP at initiation provided limited predictive value, temporal trends and individual components were strongly linked to VILI. **Conclusions**: Higher MP has been associated with adverse clinical outcomes in patients with COVID-19 receiving invasive mechanical ventilation, supporting its potential role as a prognostic indicator. Its dynamic assessment, thresholds, and integration with ventilatory strategies such as prone positioning enhance risk stratification and may guide individualized, lung-protective ventilation. Continuous monitoring and standardized calculation are recommended to optimize clinical decision-making.

## 1. Introduction

Mechanical ventilation (MV) is widely used in intensive care units to support gas exchange in patients with acute respiratory failure. However, improper adjustment of ventilatory parameters may lead to both mechanical and inflammatory lung injuries, potentially worsening the patient’s clinical condition. In this context, the concept of mechanical power (MP) offers an integrated approach to quantify and control the mechanical load imposed on the respiratory system, representing a promising tool for the individualization of lung-protective ventilation strategies [1].

The concept of MP in MV has emerged as an important marker of the impact of ventilatory forces applied to the respiratory system, as it integrates mechanical and dynamic variables into a single parameter. It is defined as the amount of energy delivered to the respiratory system per unit of time during MV, reflecting the complex interaction between pressure, tidal volume (Vt), respiratory rate, and airflow [2].

The original definition of MP proposed by Gattinoni refers to the energy transferred from the ventilator to the respiratory system only under highly controlled conditions, namely deep sedation, absence of spontaneous breathing, neuromuscular blockade, and volume-controlled ventilation [1,3]. Under these circumstances, MP does not quantify the energy delivered “to the lung”, but rather the total mechanical energy applied to the respiratory system as a whole.

In patients exhibiting spontaneous breathing activity, conventional MP calculations based solely on airway pressure and ventilator variables fail to capture the substantial portion of work generated by the respiratory muscles [4,5]. As a result, MP may underestimate true lung stress in these situations, limiting its interpretability and complicating comparisons across studies with different sedation strategies or ventilatory modes. To address these limitations, newer metrics such as compliance-normalized MP, specific MP, and transpulmonary MP have been proposed [6,7]. These approaches aim to better quantify the mechanical load applied to the lung parenchyma, particularly in patients who are not fully passive [8]. In this context, recent studies indicate that elevated MP levels are associated with an increased risk of ventilator-induced lung injury (VILI) [9].

The incidence of VILI is not well established; however, it is believed to be more frequent in patients with acute respiratory distress syndrome (ARDS) [10]. Complications resulting from VILI include pulmonary edema, barotrauma, and worsened hypoxemia. In 2016, some researchers explored the hypothesis that VILI could be explained by a single unifying variable: MP [2,11]. Pulmonary injury caused by severe acute respiratory syndrome coronavirus 2 (SARS-CoV-2) infection shares several pathophysiological features with other forms of ARDS, although it often presents initially with more severe hypoxemia and an absence of dyspnea despite evident lung injury on imaging—a phenomenon described as silent hypoxemia [12]. Patients infected with SARS-CoV-2, the virus responsible for coronavirus disease 2019 (COVID-19), may develop pulmonary inflammation may develop with fibrosis, potentially progressing to conditions that require high-energy ventilatory support. In such cases, prognosis tends to be poorer in the context of ARDS and VILI, factors that must be carefully considered in clinical management [13]. In this context, the primary goal of MV—to promote gas exchange—can be compromised when ventilator settings and mechanical resistance are inadequately managed, leading to elevated MP. This pattern has been linked to an increased occurrence of VILI and was associated with poorer clinical outcomes [13].

A study conducted in 2023 concluded that MP can serve as a relevant marker in specific clinical contexts—such as prone-position therapy in COVID-19-associated ARDS—and identified MP as an independent predictor of outcomes in this population [14]. In contrast, a secondary analysis of the CIBERESUCICOVID project—a multicenter observational cohort study conducted within the Centro de Investigación Biomédica en Red de Enfermedades Respiratorias (CIBERES), focusing on patients admitted to intensive care units (UCI: Unidad de Cuidados Intensivos) with COVID-19—reported that MP and its individual components offered limited additional value for guiding MV in the studied cohort [15]. Collectively, these findings indicate that the role of MP and its components in clinical decision-making for this patient group remains uncertain and warrants further investigation. In particular, the association between individual MP components and mortality in non–COVID-19 ARDS—especially in patients managed with lung-protective ventilation strategies—has yet to be fully elucidated.

Despite the increasing interest in MP as an integrative marker of the intensity of MV and its potential relationship with VILI, the prognostic relevance of MP in adult patients with COVID-19 undergoing invasive mechanical ventilation (IMV) has not previously been systematically synthesized. The available evidence is derived from heterogeneous observational studies that have examined different aspects of MP, including absolute values, temporal changes, individual mechanical components, cumulative exposure, and its modulation during ventilatory interventions such as prone positioning [16,17]. A comprehensive synthesis of these findings is therefore needed to clarify the current evidence regarding the association between MP and clinically relevant outcomes, including VILI, MV-related complications, and mortality in patients with COVID-19-associated acute respiratory failure.

Although the use of MP as a clinical indicator is promising, important gaps remain regarding the standardization of its calculation and its correlation with outcomes in COVID-19 patients. In this context, the present systematic review aims to synthesize the available evidence on the role of MP in patients with SARS-CoV-2 infection undergoing IMV, particularly its association with VILI and adverse clinical outcomes.

## 2. Materials and Methods

For this systematic review, the PubMed-MEDLINE (Medical Literature Analysis and Retrieval System Online) database was searched for articles published within the last five years, up to September 2024. The following search strategy was used:

[Search terms] (“Mechanical Power”) AND (“COVID-19” OR “coronavirus” OR “SARS-CoV-2” OR “SARS-CoV”) and [Filters] Humans, English language.

The detailed PubMed-MEDLINE search strategy conducted by the researchers is provided in Appendix A.

The PICOT strategy was applied as follows: (Population) adult patients with COVID-19 undergoing IMV; (Intervention) MP calculation; (Control) ventilatory parameters adjusted without MP guidance; (Outcomes) association of MP values with clinical outcomes, including VILI, complications related to MV, and mortality; (Time) studies published up to September 2024. From the selected studies, data were extracted on the impact of MP regarding (i) the incidence of VILI, (ii) complications associated with IMV, and (iii) overall clinical outcomes.

In the initial search retrieved 356 articles. Case reports, review articles, meta-analyses, and letters to the editor were excluded. After these exclusion, ten articles remained and were reviewed in full.

**Figure 1:**
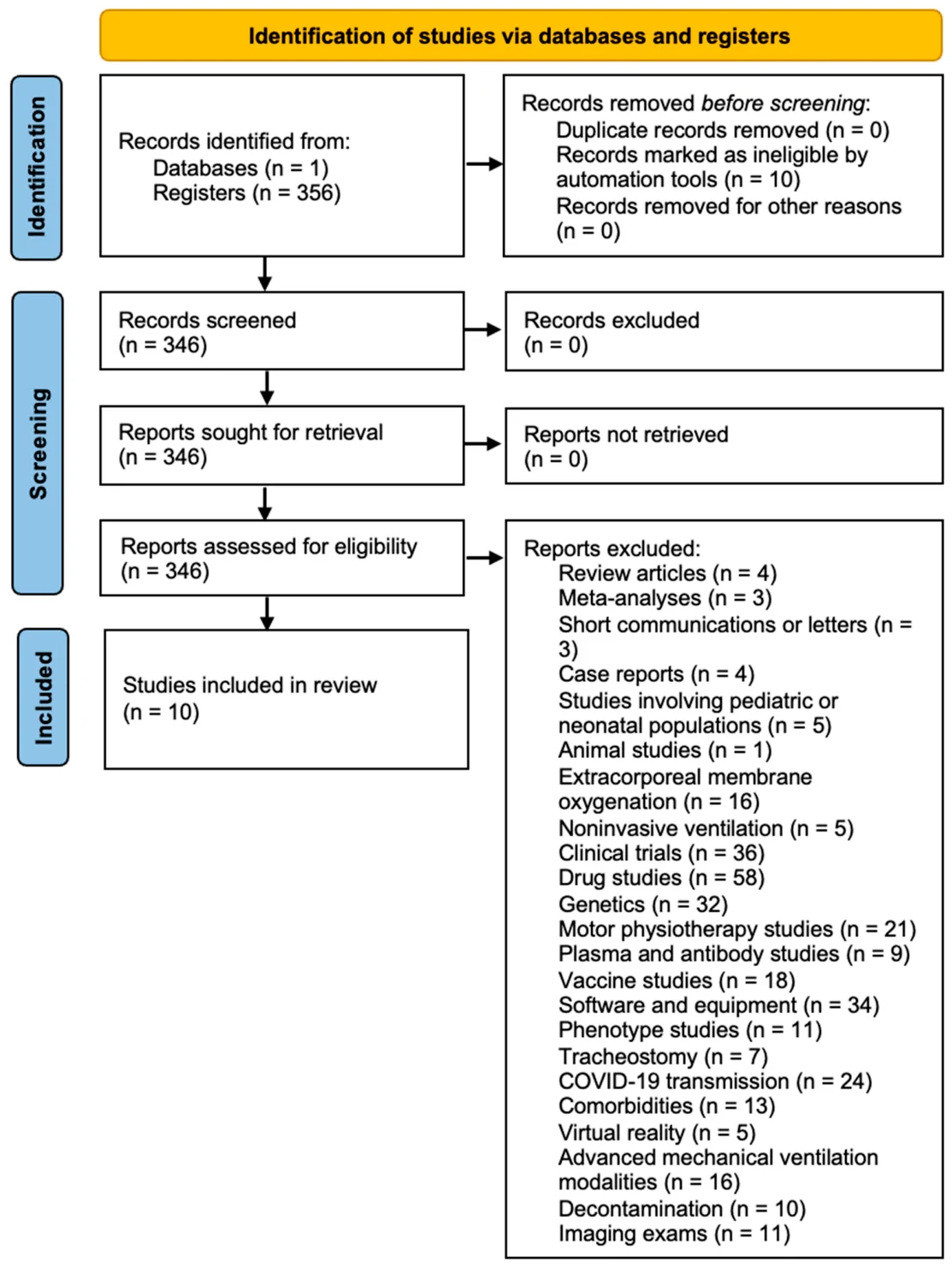
PRISMA (Preferred Reporting Items for Systematic Reviews and Meta-Analyses) flow diagram of study selection. A total of 356 articles were identified in PubMed-MEDLINE (Medical Literature Analysis and Retrieval System Online). After screening and applying the predefined inclusion and exclusion criteria, 346 studies were excluded for reasons such as study design, population, intervention, or outcomes. Finally, ten studies met the eligibility criteria and were included in the systematic review. n: number of articles.

Protocol and registration: This systematic review was not prospectively registered, and no review protocol was registered prior to the conduct of the study. The review was conducted and reported in accordance with the Preferred Reporting Items for Systematic Reviews and Meta-Analyses (PRISMA) 2020 Statement. The PRISMA 2020 flow diagram is presented as Figure 1, and the completed PRISMA 2020 Checklist is provided as supplementary material (Table S1).

In addition, the mathematical equations used to calculate MP in the included studies were extracted and summarized to facilitate comparison among the different calculation approaches. The extracted equations were organized according to the available ventilatory variables and respiratory mechanics parameters required for their application, including Vt, respiratory rate, airway pressures, respiratory system compliance, resistance, and inspiratory-to-expiratory time ratio.

## 3. Results

A total of 356 articles were initially retrieved from the PubMed-MEDLINE database using the predefined search strategy (Figure 1). After applying the inclusion and exclusion criteria, 346 studies were excluded for the following reasons: review articles (n = 4), meta-analyses (n = 3), short communications or letters (n = 3), case reports (n = 4), studies in pediatric or neonatal populations (n = 5), animal studies (n = 1), extracorporeal membrane oxygenation (n = 16), noninvasive ventilation (n = 5), clinical trials (n = 36), and other reasons related to lack of relevance to the study objectives (n = 269), including drug studies (n = 58), genetics (n = 32), motor physiotherapy (n = 21), plasma and antibody studies (n = 9), vaccine studies (n = 18), software and equipment (n = 34), phenotype studies (n = 11), tracheostomy (n = 7), COVID-19 transmission (n = 24), comorbidities (n = 13), virtual reality (n = 5), advanced ventilatory modalities (n = 16), decontamination (n = 10), and imaging exams (n = 11). After this screening process, 10 studies met the eligibility criteria and were included in the systematic review for full-text assessment.

Ten studies were included in this systematic review. Table 1 provides a summary of their main characteristics, including reference, title, year of publication, journal, and study design. Most studies were retrospective and observational, with publication dates ranging from 2021 to 2024, and all focused on critically ill patients with COVID-19 undergoing IMV.

Table 2 and Table 3 provide a comprehensive description of the objectives, methodologies, inclusion and exclusion criteria, data collection procedures, results, and conclusion of the included studies. Franck et al. (2022) investigated the influence of MP and its components on ventilatory parameters in COVID-19 patients with moderate ARDS [13]. Using two different formulas (Gattinoni-S and Giosa), they demonstrated strong correlations between MP and elastance, driving pressure (ΔP), and positive end-expiratory pressure (PEEP), highlighting the importance of calculation methods for MP interpretation [13]. Ghiani et al. (2023) analyzed prolonged MV in tracheostomized COVID-19 patients compared with those with respiratory failure from other etiologies [18]. Although MP and ventilatory ratio were initially higher in COVID-19 patients, this group showed lower weaning failure rates, suggesting disease-specific differences in respiratory mechanics [18].

González-Castro et al. (2023) applied Bayesian statistical analysis to test the hypothesis of a 17 J/min MP threshold [19]. Their findings indicated a strong association between higher MP values and 28-day mortality, reinforcing the prognostic significance of MP during the first 24 h of invasive ventilation [19]. Stalla et al. (2023) studied mechanically ventilated patients with COVID-19 ARDS in the prone position [14]. They observed that MP did not differ significantly at the beginning of prone positioning but was markedly lower among survivors at the end of the intervention, suggesting that MP trends may serve as a dynamic marker of outcome [14].

Becker et al. (2021) performed a case–control study comparing COVID-19-related ARDS with ARDS from other causes [20]. Their results revealed higher MP and altered pulmonary hemodynamics in COVID-19, pointing to disease-specific ventilatory characteristics [20]. Azizi et al. (2023) conducted a hospital registry study and confirmed that elevated MP was consistently associated with 30-day mortality, regardless of COVID-19 diagnosis, while respiratory rate emerged as a major determinant of mortality risk [21].

Aşar et al. (2024) introduced novel oxygenation and saturation indices in addition to MP, showing that new indices such as oxygen saturation index-MP and oxygen index-MP had high predictive value for intensive care unit mortality, reinforcing MP as a complementary prognostic tool [22]. The PRoVENT-COVID (The PRactice of VENTilation in COVID-19 Study) analyses by Schuijt et al. (2021a; 2021b) further strengthened these findings, demonstrating that exposure to higher MP (>17 J/min) and ventilation intensity during the first days of invasive ventilation was strongly associated with increased 28-day mortality, independent of baseline hypoxemia [23,24].

Finally, Manrique et al. (2024) used continuous real-world intensive care unit data from thousands of patients and identified a critical MP threshold around 18 J/min [25]. Not only the magnitude but also the cumulative exposure time above this threshold significantly increased intensive care unit mortality, underlining the clinical utility of real-time MP monitoring and its potential for early intervention [25].

The included studies used different mathematical approaches to calculate MP. The equations were based on distinct combinations of ventilatory and respiratory mechanics variables, including Vt, respiratory rate, airway pressures, compliance, resistance, and inspiratory-to-expiratory time ratio. The equations identified in the included studies, together with the principal variables required for their application, are summarized in Table 4.

Together, these studies reveal a consistent pattern: higher MP values have been associated with worse outcomes in COVID-19 patients under invasive ventilation. Differences between methodologies and ventilatory strategies (e.g., prone positioning, prolonged weaning, or registry-based analyses) suggest that while MP has been associated with mortality, its dynamic behavior, thresholds, and prognostic strength may vary depending on patient characteristics and clinical settings.

**Figure 2:**
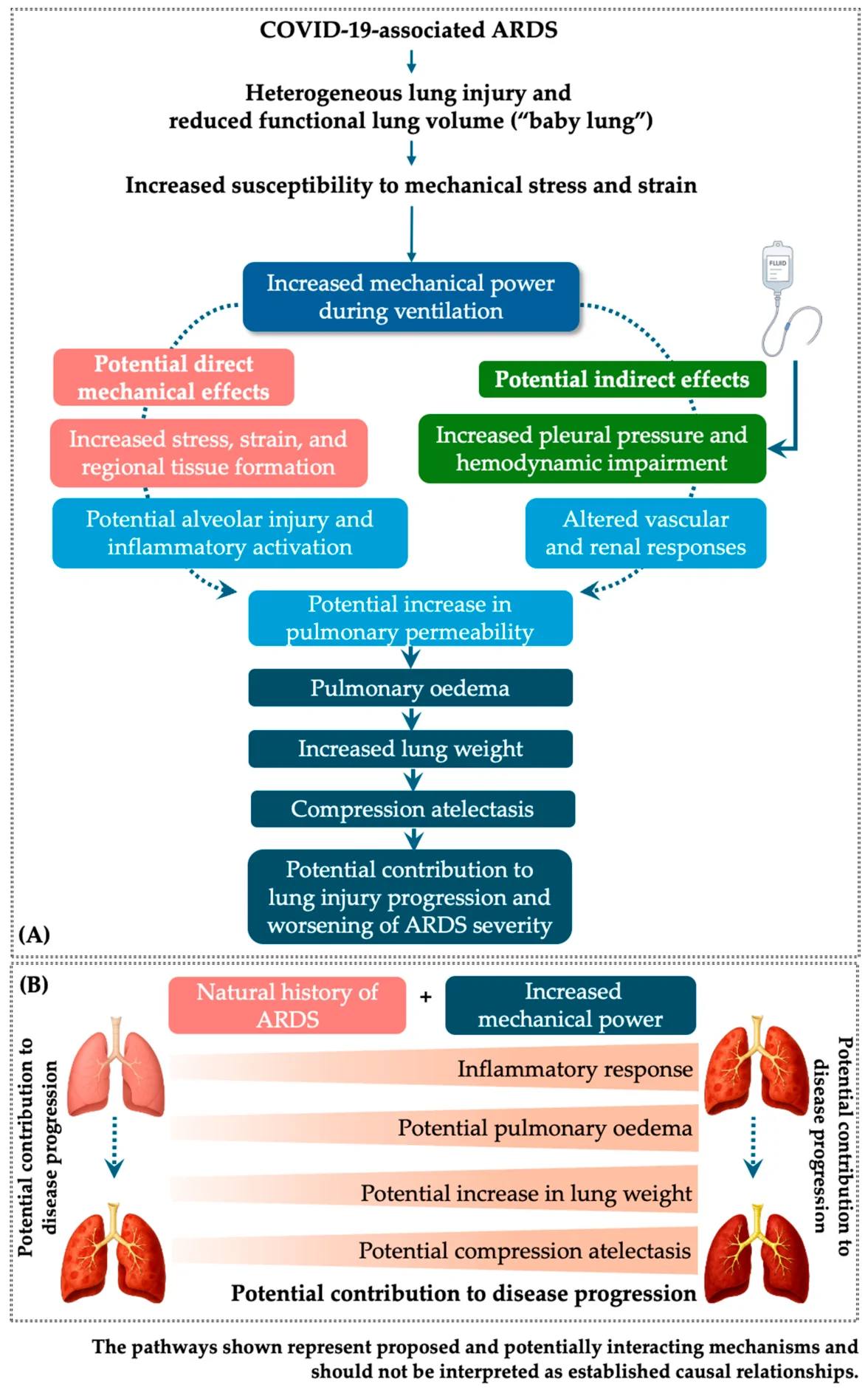
Proposed pathophysiological framework illustrating the potential contribution of mechanical power (MP) to lung injury progression in coronavirus disease 2019 (COVID-19)-associated acute respiratory distress syndrome (ARDS). (**A**) In COVID-19-associated ARDS, heterogeneous lung injury and reduced functional lung volume (“baby lung”) may increase susceptibility to ventilator-delivered mechanical energy. Increased MP (expressed in joules per minute [J/min]) may contribute to both direct mechanical effects, including increased stress, strain, regional tissue deformation, alveolar injury, and inflammatory activation, and indirect effects, such as pleural pressure changes and hemodynamic impairment leading to altered vascular and renal responses. These mechanisms may increase pulmonary permeability, favor pulmonary oedema, increase lung weight, promote compression atelectasis, and potentially contribute to lung injury progression and worsening of ARDS severity. (**B**) Conceptual representation of the potential interaction between the natural history of ARDS and increased MP, illustrating how ventilator-delivered energy may amplify inflammatory responses, pulmonary oedema, increased lung weight, and compression atelectasis, thereby potentially contributing to disease progression. The pathways illustrated represent proposed pathophysiological mechanisms and should not be interpreted as established causal relationships.

Importantly, the systemic vascular and hemodynamic disturbances associated with severe COVID-19 may persist beyond the acute respiratory phase, providing a potential biological link between acute cardiopulmonary stress and the endothelial, vascular, myocardial, and thrombotic abnormalities reported in post-COVID-19 conditions. In brief, the pathophysiological mechanisms of lung injury and compression atelectasis induced by COVID-19 and exacerbated by MP are shown in Figure 2.

Importantly, the direct and indirect pathways illustrated in Figure 2 may extend beyond the acute pulmonary consequences of MV. In severe COVID-19, SARS-CoV-2-associated endothelial dysfunction, systemic inflammation, microvascular injury, and hemodynamic disturbances may interact with the mechanical stresses imposed by ventilation. Through the direct pathway, excessive MP may amplify alveolar and epithelial injury, increase pulmonary vascular permeability, and promote inflammatory signaling, potentially contributing to systemic endothelial and microvascular dysfunction. Through the indirect pathway, increased intrathoracic pressure and hemodynamic impairment may reduce venous return, alter cardiac loading conditions, compromise organ perfusion, and activate renal and neurohumoral mechanisms that promote sodium and water retention, thereby aggravating pulmonary edema and atelectasis. These acute pulmonary, vascular, and hemodynamic disturbances may have consequences beyond the acute respiratory phase.

Recent evidence also indicates that post-COVID-19 cardiovascular sequelae may include subclinical myocardial dysfunction, arrhythmias, endothelial injury, increased arterial stiffness, elevated cardiac biomarkers, new-onset hypertension, and persistent electrocardiographic abnormalities, including in individuals without previous cardiovascular disease [19]. Proposed mechanisms include persistent endothelial dysfunction, chronic inflammation, dysregulation of the renin–angiotensin–aldosterone system, autonomic imbalance, and a prothrombotic state. Thus, the mechanisms depicted in Figure 2 may represent part of a broader pathophysiological continuum linking acute pulmonary and systemic stress with persistent cardiovascular abnormalities after SARS-CoV-2 infection. However, the current evidence does not establish a direct causal relationship between MP during invasive ventilation and long-term cardiovascular sequelae or long COVID. Rather, excessive ventilatory energy may amplify the acute cardiopulmonary, endothelial, and systemic stress associated with severe COVID-19, potentially contributing to the biological context in which persistent organ dysfunction develops.

Although the available evidence does not establish that MP independently causes long-term cardiovascular sequelae or long COVID, the amplification of acute cardiopulmonary and endothelial stress by excessive ventilatory energy represents a biologically plausible mechanism that warrants further investigation.

## 4. Discussion

This systematic review synthesized recent studies investigating the influence of MP on clinical outcomes in patients with acute respiratory failure, with a particular focus on those affected by COVID-19. The included studies examined the individual components of MP and their roles across different clinical scenarios, including prolonged MV, prone positioning, and comparisons with other etiologies of respiratory failure. Collectively, these findings support the hypothesis that MP functions as an integrative parameter capable of quantifying the total mechanical load applied to the respiratory system. It encompasses key ventilatory variables such as Vt, ΔP, respiratory rate, and PEEP, thereby providing a comprehensive assessment of the ventilatory burden. This interpretation aligns with the pathophysiological model proposed by Gattinoni et al. (2016) [2].

The concept of MP synthesizes in a single metric the key determinants of MV, including Vt, respiratory rate, flow, and pressure, which together define the amount of energy transferred to the respiratory system. Unlike the interpretation of these variables in isolation, MP provides a physiologically coherent framework that integrates the mechanisms underlying VILI, namely barotrauma, volutrauma, and atelectrauma, thereby overcoming the limitations of assessing individual parameters alone [9].

VILI is a well-recognized complication of MV, particularly in patients with acute respiratory failure or ARDS. It results from the interaction of mechanical forces imposed by the ventilator with the intrinsic fragility of the injured lung. Several ventilatory parameters, when inadequately set, play a central role in triggering or amplifying VILI through different mechanisms. One of the most critical parameters is the Vt. Excessive Vt, particularly above 6–8 mL/kg of predicted body weight, causes overdistension of alveoli, leading to volutrauma. This mechanical overstretch disrupts alveolar epithelial and endothelial integrity, increases vascular permeability, and promotes inflammatory mediator release. Similarly, plateau pressure (Pplat) reflects the static pressure exerted on the alveoli at the end of inspiration. When Pplat exceeds 30 cmH_2_O, the risk of barotrauma rises substantially, resulting in alveolar rupture, pneumothorax, or interstitial emphysema. Closely related to these parameters is ΔP, defined as the difference between Pplat and PEEP. ΔP has emerged as one of the strongest predictors of mortality in ARDS, with values above 15 cmH_2_O being associated with worse outcomes, as it represents the cyclic stress applied to the lungs with each breath [26,27,28,29].

A further challenge in adapting lung-protective ventilation is that respiratory-system compliance may not adequately reflect the mechanical heterogeneity of the underlying lung parenchyma. Pre-existing or concomitant pulmonary lesions may coexist with diffuse inflammatory injury and can substantially alter baseline respiratory mechanics, regional compliance, and the distribution of ventilatory stress. For example, pulmonary malignancies may be accompanied by additional malignant or nonmalignant lesions that are difficult to distinguish preoperatively and may coexist within the same lung, illustrating the structural complexity that may influence the interpretation of respiratory mechanics [30]. In such settings, a single global compliance value may conceal substantial regional differences in tissue stiffness, aeration, recruitability, and susceptibility to overdistension or cyclic collapse.

Importantly, pulmonary heterogeneity is also a characteristic feature of COVID-19-related lung injury [31]. The extent and distribution of pulmonary involvement may vary considerably among patients, resulting in substantial differences in lung aeration, recruitability, and response to ventilatory interventions [32,33,34]. Consequently, similar values of global respiratory-system compliance may not necessarily indicate comparable regional lung mechanics or an equivalent response to PEEP. Therefore, ventilatory management should not rely exclusively on global respiratory-system compliance but should integrate the patient’s clinical condition, gas-exchange profile, lung morphology, recruitability, and response to ventilatory adjustments to support individualized lung-protective ventilation.

This heterogeneity reinforces the importance of interpreting compliance, ΔP, Vt, PEEP, and MP within the context of the underlying pulmonary structure and disease trajectory, rather than applying fixed thresholds in isolation. This consideration is particularly relevant when MP is used as an integrative marker, because the same absolute energy load may have different biological consequences depending on the amount, distribution, and mechanical properties of the aerated lung available to receive that energy. Therefore, although global compliance remains clinically useful, it should be interpreted as an aggregate measure that may obscure regional mechanical heterogeneity. This limitation is especially important in complex pulmonary diseases, in which pre-existing lesions, superimposed inflammatory injury, fibrosis, or other structural abnormalities may alter baseline respiratory mechanics and modify the relationship between ventilatory energy, tissue stress, and VILI.

The adjustment of PEEP itself is a double-edged sword. Insufficient PEEP leads to repetitive opening and closing of alveoli during tidal breathing, a phenomenon known as atelectrauma, which exacerbates shear stress at the alveolar–capillary interface. On the other hand, excessive PEEP may cause hyperinflation of already open lung units, resulting in additional mechanical strain and hemodynamic compromise. Thus, finding the optimal PEEP remains a crucial element of protective ventilation [35,36].

Respiratory rate is another determinant of injury. High respiratory rates amplify the cumulative mechanical energy delivered to the lungs, thereby increasing the risk of VILI, even if individual tidal breaths appear protective. This cumulative energy load has been conceptualized in terms of MP, a variable that integrates Vt, ΔP, respiratory rate, PEEP, and inspiratory flow into a single measure of the energy delivered to the respiratory system per unit of time. Elevated MP has been consistently associated with worse outcomes, including prolonged MV, weaning failure, and higher mortality [9].

Flow settings also influence the risk of lung injury. Excessive or abrupt inspiratory flow patterns can impose additional stress on alveolar units, while inappropriate flow delivery may worsen patient-ventilator asynchrony, further contributing to injury. Similarly, elevated mean airway pressure, often resulting from high PEEP levels or prolonged inspiratory times, can affect not only lung mechanics but also intrathoracic hemodynamics, reducing venous return and cardiac output while promoting pulmonary vascular congestion [37].

Altogether, these parameters demonstrate that VILI is not caused by a single factor but rather by the complex interplay of ventilatory settings and lung mechanics. Volutrauma, barotrauma, and atelectrauma represent the classic components of mechanical injury, while biotrauma—resulting from the release of pro-inflammatory mediators triggered by mechanical stress—links local alveolar injury to systemic consequences such as multiorgan dysfunction. Among the different approaches to understanding VILI, MP has gained attention as it integrates the contribution of Vt, ΔP, respiratory rate, PEEP, and flow into a unifying concept. By quantifying the energy burden imposed on the lungs, MP offers a more physiologically meaningful predictor of damage potential than isolated parameters. In this context, VILI arises from the inappropriate application of Vt, Pplat, ΔP, PEEP, respiratory rate, and inspiratory flow, all of which contribute to excessive MP delivered to the lungs. Careful adjustment of these parameters through lung-protective strategies is essential to minimize alveolar stress and strain, reduce systemic inflammation, and ultimately improve outcomes in critically ill patients, particularly those with ARDS and COVID-19 who are most vulnerable to the cumulative impact of prolonged IMV [9].

In the setting of COVID-19, this approach becomes particularly relevant. Patients with severe disease frequently present with ARDS, characterized by heterogeneous alterations in lung mechanics, prolonged dependence on IMV, and a heightened cumulative risk of pulmonary damage. Traditional indices such as ΔP or PEEP, although valuable, may fail to capture the overall energy burden imposed on the lung tissue during prolonged ventilation. By encompassing the total energy delivered to the respiratory system, MP offers a more comprehensive and realistic measure of the injurious potential of ventilatory strategies in this specific context [9,38].

Emerging evidence suggests that elevated MP levels are associated with worse outcomes in critically ill COVID-19 patients, including prolonged ventilation, higher rates of weaning failure, and increased mortality. This recognition has stimulated growing interest in MP not only as a prognostic indicator but also as a potential therapeutic target for guiding ventilatory adjustments. By tailoring ventilatory support to minimize excessive MP, clinicians may reduce the risk of VILI and improve outcomes in this highly vulnerable population [13,21].

The IMV, while indispensable as a life-sustaining intervention, carries inherent risks, among which VILI is one of the most extensively documented and studied. For decades, research has examined the role of energy transfer to the lungs during ventilatory support in the pathogenesis of VILI. Within this context, MP has emerged as a comprehensive parameter that integrates all ventilatory factors contributing to lung injury. Elevated MP values have been associated with increased incidence of VILI, longer intensive care unit stays, and prolonged duration of IMV [39,40].

COVID-19 may progress to ARDS, a condition characterized by heterogeneous lung parenchyma, regional disparities in ventilation, and altered respiratory mechanics [12]. The management of patients requiring IMV is particularly challenging and is often associated with poor outcomes, including high mortality rates [41]. Within this framework, MP has been recognized as a comprehensive physical parameter that integrates all components contributing to VILI [9]. Moreover, MP has been described as an independent risk factor associated with mortality in critically ill patients [42].

A retrospective observational cohort study including 249 mechanically ventilated and tracheostomized patients with and without COVID-19–related respiratory failure evaluated the trajectories of pulmonary dead space fraction and MP during weaning [18]. Secondary outcomes included weaning failure rates between groups and the predictive ability of pulmonary dead space fraction and MP for weaning outcomes [18]. Patients with COVID-19 exhibited higher values for both indices throughout the weaning process: ventilatory ratio and MP at initiation, and ventilatory ratio and MP at completion [18]. The predictive capacity of MP for weaning success or failure varied according to lung and chest wall compliance, with COVID-19 patients consistently demonstrating greater dynamic compliance and fewer weaning failures (9% vs. 30%) [18]. These findings indicate that COVID-19 patients differ in ventilatory efficiency and respiratory mechanics compared to other long-term ventilated individuals, presenting with higher ventilatory ratio and MP values. Differences in MP were attributed to greater lung–chest wall compliance in COVID-19 patients, which may have contributed to the lower weaning failure rate observed [18]. In this study, both groups were classified as prolonged weaning cases. According to current evidence, patients undergoing prolonged weaning typically present with complex pathophysiology and require a multidisciplinary approach, as the underlying causes are diverse and must be addressed beyond correction of the initial insult leading to respiratory failure [43].

A 2023 study reported significant differences in MP measurements between survivors and non-survivors with ARDS requiring prone positioning, but only during the final prone session [14]. This difference was primarily driven by an increase in MP among non-survivors (baseline difference = 3.63 J/min; 95% CI = 0.31–6.94), which was not observed in survivors (baseline difference = 0.02 J/min; 95% CI = −2.66 to 2.70) [14]. In multivariable analysis, MP remained independently associated with higher hospital mortality after adjustment for relevant covariates, including the Simplified Acute Physiology Score III (SAPS III), duration of MV, patient age, and number of prone sessions [14]. These findings suggest that MP acted as an independent predictor of mortality in patients with COVID-19–related ARDS managed with prone positioning.

Conversely, a multicenter observational cohort study of mechanically ventilated patients with COVID-19 investigated the associations between MP and its individual components—static elastic, dynamic elastic, total elastic, and resistive power—with mortality, particularly at 90 days. The main findings were as follows: (i) at the initiation of MV, neither MP nor any of its components were independently associated with mortality; and (ii) after three days of ventilation, although higher static and total elastic power were associated with reduced survival, simpler variables such as PEEP and Pplat captured this increased risk in a comparable manner. These results suggest that MP and its components may provide only limited additional value in guiding ventilatory management [15]. Overall, the findings highlight the need for further investigation into the role of MP and its components in clinical decision-making, particularly regarding their relationship with mortality in non–COVID-19 ARDS patients, especially those managed with lung-protective ventilation strategies.

Additionally, a recent analysis from a prospective cohort study in patients with COVID-19 demonstrated that early initiation of prone positioning (within 48 h of MV onset) was associated with reduced mortality, underscoring the critical importance of timing in this intervention [44]. Furthermore, an observational study of 22 intubated patients who underwent prone positioning, which combined measurements of respiratory compliance with regional distribution of ventilation, revealed that only a subset of patients experienced improved compliance and effective lung recruitment following prone positioning, whereas others maintained or even exhibited reduced compliance. This finding highlights the heterogeneity of the mechanical response to prone positioning [45]. Collectively, these studies suggest that although MP represents a valuable parameter for monitoring and risk stratification in ARDS, its clinical utility is maximized when integrated with dynamic variables such as timing of intervention, elastic components, and individual patient characteristics under lung-protective ventilation strategies. Such integration is essential for optimizing ventilatory management and minimizing the risk of VILI.

An observational study demonstrated that elevated MP values—particularly those driven by higher Vt and ΔP—were correlated with unfavorable outcomes [13]. This association is consistent with findings reporting that higher MP values during the first 24 h of MV were independently associated with increased 30-day mortality [21]. Collectively, these studies reinforce the importance of an individualized approach to MV, in which continuous monitoring of MP and its components may guide safer and more effective ventilatory strategies, thereby reducing the risk of VILI and improving clinical outcomes in patients with COVID-19-related ARDS. These findings have also been corroborated by other investigations, supporting the hypothesis that MP may serve not only as a marker of disease severity but also as a potential therapeutic target for lung-protective ventilation strategies.

An observational study sought to evaluate the evidentiary strength of statistical hypotheses regarding 28-day mortality and the proposed threshold of 17 J/min for MP in patients with respiratory failure due to SARS-CoV-2 infection. The findings indicated that higher MP values during the first 24 h of MV were associated with worse prognosis in patients both with and without COVID-19 19 [19]. These results are consistent with those of a multicenter cohort including 553 mechanically ventilated patients, which demonstrated a significant association between increases in MP and 28-day mortality. Furthermore, the study highlighted the importance of continuous monitoring and adjustment of ventilatory parameters in response to patients’ clinical evolution, emphasizing that variations in MP provide additional value for risk stratification and prognostic assessment [24].

An observational case–control study involving patients with respiratory failure due to COVID-19 investigated pulmonary ventilatory and hemodynamic differences between individuals with COVID-19-induced ARDS and those with ARDS from other causes. Based on physiological and hemodynamic parameters, the study revealed that, despite respiratory compliance comparable to non-COVID ARDS patients, MP was higher, indicating early microvascular dysfunction and altered right ventricle–pulmonary coupling. The authors concluded that the MP applied to the lungs of COVID-19 ARDS patients through MV was greater than that observed in ARDS from other etiologies [20].

Complementing these findings, a cohort study of 343 patients by Asar and colleagues assessed indices based on the ratio of peripheral oxygen saturation to fraction of inspired oxygen (SpO_2_/FiO_2_) and the ratio of arterial partial pressure of oxygen to fraction of inspired oxygen (PaO_2_/FiO_2_, P/F). Intensive care unit mortality rates were higher among patients whose P/F and P/F × PEEP ratios fell below identified cutoff values. Additionally, patients exhibiting elevated MP experienced increased intensive care unit mortality, whereas those with MP below the thresholds demonstrated more favorable outcomes. The study concluded that these indices performed comparably to conventional measures in predicting clinical progression, highlighting their potential as complementary monitoring tools in ARDS patients [22]. Furthermore, a retrospective analysis of 2623 patients requiring IMV for more than 24 h, with automated data collection every two minutes, demonstrated that each hour spent with MP exceeding 18 J/min increased the likelihood of intensive care unit mortality by approximately 0.3% [25].

Collectively, the recent literature underscores that, although COVID-19 exhibits distinct hemodynamic features, the prognostic relevance of MP is supported by quantitative evidence. These findings suggest that ventilatory optimization in COVID-19-related ARDS should account not only for MV parameters but also for hemodynamic assessment.

In severe cases of COVID-19 complicated by ARDS, the lung is markedly heterogeneous, with regions of variable compliance. This heterogeneity means that the same value of MP may result in different energy densities across the ventilated lung parenchyma. Because ventilation is preferentially distributed to the most fragile or overdistensible regions, energy delivery tends to concentrate in these vulnerable zones, thereby increasing the likelihood of VILI. In this context, reducing the total energy delivered per minute, and particularly limiting its concentration within the functional lung, may represent a relevant strategy for mitigating the risk of VILI. However, the clinical effects of reducing mechanical energy exposure in patients with COVID-19 remain dependent on individual lung characteristics and require further investigation [5].

The application and interpretation of MP in COVID-19-related ARDS are complicated by the marked heterogeneity of the disease [46]. One proposed contributor to this heterogeneity is the presence of distinct patterns of respiratory-system mechanics, characterized by differences in compliance and elastance, as described by Gattinoni et al. and initially conceptualized as the “L” and “H” phenotypes [31,47]. The “L” phenotype is characterized by low elastance, relatively preserved compliance, and low potential for recruitment, whereas the “H” phenotype is characterized by high elastance, reduced compliance, and greater potential for recruitment. Subsequent studies evaluating these proposed phenotypes in patients with COVID-19 confirmed substantial interindividual variability in compliance and elastance, which may influence the response to MV and change over time as the disease progresses [48,49].

The COVID-19 pandemic has underscored the urgent need for individualized ventilatory management aimed at preventing alveolar damage [50,51,52]. Among the key lessons learned are (i) the highly heterogeneous nature of ARDS lungs; (ii) the necessity of tailoring PEEP values according to recruitability rather than fixed thresholds; and (iii) the importance of continuous evaluation of elastic components such as Vt, airway pressure, and flow. Within this framework, MP provides a comprehensive summary of ventilatory intensity, condensing multiple parameters into a single physiologic quantity and thereby facilitating sequential titration of ventilatory targets [2,5,24].

Respiratory system mechanics in COVID-19 are dynamic and may change substantially over the course of the disease, particularly with respect to compliance and elastance [47,53]. These changes may reflect progressive tissue injury, patient self-inflicted lung injury resulting from excessive respiratory effort, and pulmonary edema associated with the release of inflammatory mediators. This instability requires frequent adjustments to ventilator settings, which may lead to changes in absolute MP values throughout the course of ventilatory support. Furthermore, patients with COVID-19 are often managed with higher respiratory rates to increase minute ventilation and reduce hypercapnia, a ventilatory variable that directly contributes to increased MP. In addition, most studies evaluating the behavior of MP in patients with COVID-19 included in this review were observational, making it difficult to determine whether increased MP is a causal contributor to lung injury or primarily a marker of disease severity [54].

Emerging research has explored the use of MP in protocolized strategies, in which ventilatory adjustments are guided by energy thresholds rather than isolated parameters. Studies evaluating MP personalization and its integration with imaging techniques, such as computed tomography or lung ultrasound, suggest that aligning ventilatory targets with the degree of lung heterogeneity may optimize alveolar protection. This approach highlights the potential clinical relevance of MP-guided ventilation by reducing unnecessary mechanical energy exposure, particularly in patients with severe COVID-19-associated ARDS [9]. This approach highlights the potential clinical relevance of MP-guided ventilation by reducing unnecessary mechanical energy exposure, particularly in patients with severe COVID-19-associated ARDS. However, the available evidence is primarily observational, and reported associations between lower MP and improved survival should not be interpreted as evidence of a causal benefit. Prospective studies are needed to determine whether ventilation strategies specifically targeting lower MP can improve clinical outcomes.

Nevertheless, despite its promise, the use of MP as a routine bedside tool requires caution. Its limitations include variability in calculation formulas, dependence on accurate measurement of ΔP and flow, and the lack of a universally accepted threshold to guide clinical practice. Although MP provides an integrative measure of the energy transferred from the ventilator to the respiratory system per unit of time—thus enabling a more precise assessment of ventilation intensity and the risk of VILI—it has not yet achieved universal consensus as a standard clinical parameter. The absence of clearly defined cutoffs for “safe” versus “injurious” levels of MP continues to limit its translation into daily practice, underscoring the need for prospective validation studies and consensus guidelines before broad implementation [55,56].

The findings reported by Manrique et al. further reinforce the importance of considering MP as a dynamic parameter rather than as an isolated baseline measurement [25]. Collectively, these data suggest that changes in MP may provide clinically relevant information regarding disease progression and response to ventilatory management. However, whether MP-guided interventions can improve patient outcomes remains uncertain and requires confirmation in well-designed prospective studies.

Future investigations should prioritize the evaluation of MP within the context of the heterogeneous realities of intensive care units ICUs, acknowledging the substantial variability in patient characteristics, resource availability, ventilatory practices, and healthcare system capacity across institutions and countries [57,58,59]. This perspective is particularly relevant during pandemic scenarios, in which unprecedented surges of critically ill patients place extraordinary pressure on healthcare infrastructures, potentially influencing ventilatory management strategies and clinical outcomes. Accordingly, large-scale international multicenter collaborative studies, similar to the GlobalSurg and COVIDSurg initiatives, should be encouraged to investigate the prognostic significance and clinical applicability of MP across diverse intensive care unit settings [60,61,62,63,64]. Such collaborative efforts would provide robust and generalizable evidence regarding the relationship between ventilator-delivered mechanical energy and clinically relevant outcomes, not only in patients with COVID-19-associated ARDS but also across a broader spectrum of acute respiratory failure and critical illness. Ultimately, these investigations may facilitate the establishment of standardized, evidence-based MP-guided ventilatory strategies that are adaptable to the diversity of contemporary critical care practice while improving patient outcomes.

### 4.1. Limitations

Although MP is a promising metric for assessing ventilation intensity and the risk of VILI, its implementation during the pandemic has faced several challenges. These include the absence of consensus on optimal target ranges, variability in the components and formulas used to calculate MP, and the need for further clinical validation to support routine adoption. Additional limitations must also be considered: cohort heterogeneity restricts generalizability, methodological inconsistencies in MP definitions complicate comparisons across studies, and most available evidence derives from observational designs, which are susceptible to residual confounding and limit causal inference. Together, these factors underscore the need for a cautious, evidence-based approach when applying MP in clinical practice.

A further limitation relates to the variable presence of spontaneous breathing activity across the included studies. The original definition of MP assumes a fully passive patient under deep sedation, neuromuscular blockade, and volume-controlled ventilation. Under these conditions, MP reflects only the energy delivered by the ventilator to the respiratory system. In contrast, patients exhibiting spontaneous breathing generate a substantial portion of the total mechanical energy through respiratory muscle effort. Conventional MP calculations, which rely exclusively on airway pressure and ventilator variables, do not capture this patient-generated work. Consequently, MP may underestimate true lung stress in non-passive patients, particularly during assisted ventilation or prolonged weaning. This variability in sedation depth, ventilatory mode, and neuromuscular blockade protocols complicates comparisons between studies and may partly explain differences in reported associations between MP and clinical outcomes.

This issue is especially relevant in COVID-19 ARDS, where vigorous inspiratory effort may contribute to patient self-inflicted lung injury, amplifying transpulmonary pressure swings and increasing the risk of VILI. Studies that did not report sedation depth, neuromuscular blockade use, or the degree of spontaneous breathing may therefore have underestimated the actual mechanical load applied to the lung parenchyma. To address these limitations, newer metrics such as compliance-normalized MP, specific MP, and transpulmonary MP have been proposed. These approaches aim to better quantify the mechanical load applied to the respiratory system, particularly in non-passive patients or in conditions of heterogeneous aeration. However, these advanced metrics were not available in most of the studies included in this review.

Finally, the available evidence provides limited information regarding how pre-existing or concomitant structural pulmonary abnormalities may influence the interpretation of MP and its relationship with tissue stress. Global respiratory-system compliance may not fully capture regional mechanical heterogeneity in lungs affected by overlapping lesions, fibrosis, heterogeneous aeration, or superimposed inflammatory injury. Consequently, the same absolute MP may have different biological consequences depending on the amount, distribution, and mechanical properties of the aerated lung receiving the delivered energy. In addition, most included studies focused on short-term outcomes during the acute phase of COVID-19-related ARDS, and data linking MP exposure during invasive ventilation with persistent systemic, cardiovascular, or other post-acute sequelae remain scarce. Therefore, the long-term clinical implications of MP and the extent to which acute ventilatory energy exposure may interact with persistent endothelial, vascular, myocardial, or systemic abnormalities remain uncertain and warrant prospective longitudinal investigation.

An additional limitation concerns the temporal assessment of MP exposure across the included studies. Most investigations evaluated MP at a single time point or during a limited period of MV, rather than accounting for the duration, cumulative burden, or temporal trajectory of exposure. However, the biological effects of ventilator-induced energy transfer are unlikely to depend exclusively on a single absolute MP value and may instead reflect the interaction between the magnitude and duration of exposure, as well as changes in lung mechanics and disease severity over time. Consequently, isolated measurements may not adequately capture the cumulative mechanical burden experienced by the lung. Moreover, differences in the timing of MP assessment relative to intubation, disease progression, prone positioning, changes in ventilatory mode, and clinical outcomes may introduce additional sources of heterogeneity and limit direct comparisons between studies. Future prospective studies should therefore evaluate time-dependent and cumulative measures of MP exposure, including dynamic trajectories and dose–response relationships, to better define the relationship between ventilator-delivered energy and clinical outcomes.

### 4.2. Recommendations for Future Research

The findings of this systematic review identify several important gaps in the current evidence regarding the clinical relevance of MP in patients with COVID-19 undergoing IMV. The limited number of eligible studies, the predominance of observational designs, and the heterogeneity in MP calculation methods, measurement timepoints, patient populations, and clinical outcomes limit the comparability of findings and preclude the establishment of universally applicable prognostic thresholds.

Future prospective, multicenter studies should therefore use standardized methods for calculating and reporting MP. These studies should clearly specify the calculation formula used, the timing and frequency of MP measurements, the individual mechanical components contributing to MP, and the duration of exposure to elevated MP values. Such standardization would facilitate comparisons between studies and help determine whether specific MP thresholds have consistent clinical relevance across different populations.

A further important gap concerns the limited evaluation of MP as a time-dependent parameter. Future investigations should move beyond isolated measurements obtained at the initiation of MV and systematically evaluate the temporal evolution and cumulative exposure to MP throughout the course of ventilation. Repeated measurements may provide greater prognostic information than a single baseline value and may help clarify whether changes in MP reflect disease progression, response to treatment, or modifications in ventilatory management.

Future research should also further investigate the independent and potentially interacting contributions of the individual components of MP, including Vt, ΔP, respiratory rate, and PEEP. This may help determine whether the clinical relevance of MP is primarily related to the total energy delivered to the respiratory system or whether specific components are more strongly associated with adverse outcomes and may represent more appropriate therapeutic targets.

Finally, interventional studies are needed to determine whether strategies aimed at reducing MP or limiting cumulative exposure to elevated MP values can improve patient-centered outcomes, including mortality, duration of MV, and VILI. Additional studies should evaluate the behavior and prognostic relevance of MP across different clinical and respiratory phenotypes and during specific ventilatory interventions, including prone positioning. Collectively, these investigations may help determine whether MP should remain primarily a prognostic marker or can be incorporated as a clinically actionable parameter to guide individualized protective ventilation.

## 5. Conclusions

The available evidence suggests that MP is a clinically relevant integrative parameter for assessing the intensity of MV and is associated with clinically important outcomes, including VILI, prolonged MV, weaning failure, and mortality in patients with COVID-19-related acute respiratory failure. These findings support the potential role of MP as a complementary tool for risk stratification and for informing individualized protective ventilation strategies.

However, the prognostic interpretation of MP should consider its temporal evolution, the relative contribution of its individual components, the underlying pulmonary phenotype, respiratory-system mechanics, spontaneous respiratory effort, and hemodynamic status. Global compliance and conventional ventilator-derived MP calculations may not fully capture regional mechanical heterogeneity or patient-generated respiratory effort, particularly in structurally complex or non-passive lungs. Therefore, MP should currently be regarded as a complementary marker rather than an isolated therapeutic target, and its association with adverse outcomes should not be interpreted as evidence of causality.

The heterogeneity of the available evidence, including differences in patient populations, MP calculation methods, measurement timepoints, ventilatory strategies, and outcome definitions, warrants cautious interpretation of current findings. Further well-designed prospective studies using standardized and longitudinal approaches to MP assessment are needed to clarify the prognostic value of time-varying and cumulative mechanical energy exposure, establish clinically relevant thresholds, and determine whether MP-guided ventilatory strategies improve patient-centered outcomes. The potential relationship between acute ventilatory energy exposure and persistent systemic or cardiovascular sequelae after severe SARS-CoV-2 infection also remains insufficiently characterized and warrants further investigation.

## Figures and Tables

**Table 1:** Summary of the included studies: reference, title, year of publication, journal, and study design of the articles selected for this systematic review.

Title (Reference)	Year	Journal	Study Design
Influence of mechanical power and its components on mechanical ventilation in SARS-CoV-2 [13]	2022	*Revista Brasileira de Terapia Intensiva*	Observational, longitudinal, analytical, and quantitative study
Ventilatory ratio and mechanical power in prolonged mechanically ventilated COVID-19 patients versus respiratory failures of other etiologies [18]	2023	*Therapeutics Advances in Respiratory Disease*	Retrospective observational cohort study
The Bayes factor in the analysis of mechanical power in patients with severe respiratory failure due to SARS-CoV-2 [19]	2023	*Intensive Medicine*	Analytical observational cohort study
Mechanical power in prone position intubated patients with COVID-19-related ARDS: A cohort study [14]	2023	*Critical Care Research and Practice*	Retrospective single-center cohort study
Pulmonary hemodynamics and ventilation in patients with COVID-19-related respiratory failure and ARDS [20]	2021	*Journal of Intensive* *Care Medicine*	Observational case–control study
Mechanical power and 30-day mortality in mechanically ventilated, critically ill patients with and without coronavirus disease-2019: A hospital registry study [21]	2023	*Journal of Intensive Care*	Retrospective analytical study
Novel oxygenation and saturation indices for mortality prediction in COVID-19 ARDS patients: The impact of driving pressure and mechanical power [22]	2024	*Journal of Intensive* *Care Medicine*	Retrospective analytical study
Association of time-varying intensity of ventilation with mortality in patients with COVID-19 ARDS: Secondary analysis of the PRoVENT-COVID study [23]	2021	*Frontiers in Medicine*	Retrospective observational study
Association of intensity of ventilation with 28-day mortality in COVID-19 patients with acute respiratory failure: insights from the PRoVENT-COVID study [24]	2021	*Critical Care*	Secondary analysis of a retrospective multicenter observational study
Impact of mechanical power on ICU mortality in ventilated critically ill patients: a retrospective study with continuous real-life data [25]	2024	*European Journal of Medical Research*	Retrospective observational study

ARDS: acute respiratory distress syndrome, COVID-19: coronavirus disease 2019 (COVID-2019), ICU: intensive care unit, PRoVENT-COVID: The PRactice of VENTilation in COVID-19 Study, SARS-CoV-2: severe acute respiratory syndrome coronavirus 2.

**Table 2:** Description of the objectives, inclusion and exclusion criteria, and methodology (interventions and data collection) of the studies included in this systematic review.

Ref	Objectives	Methods	Inclusion/Exclusion Criteria	Interventions and Data Collection
[13]	To analyze the influence of mechanical power (MP) and its components in the mechanical ventilation (MV) of patients with coronavirus disease 2019 (COVID-19), identifying their values, correlations, and the effects on the outcomes of the Gattinoni-S and Giosa formulas.	This is an observational, longitudinal, and analytical study of MP and ventilatory parameters in patients with severe acute respiratory syndrome coronavirus 2 (SARS-CoV-2) admitted to the intensive care unit (ICU) of a university hospital between March and May 2021. A total of 150 patients with moderate acute respiratory distress syndrome (ARDS) were included, all under volume-controlled ventilation, deep sedation, and neuromuscular blockade.	Inclusion criteria: World Health Organization (WHO) severity scale (six or seven), ratio of partial pressure of arterial oxygen to the fraction of inspired oxygen (PaO_2_/FiO_2_) between 100 and 200, chest X-ray or computed tomography (CT) scan with bilateral opacities, normal D-dimers, and reverse transcription polymerase chain reaction (RT-PCR) positive for SARS-CoV-2.	A total of 150 patients diagnosed with COVID-19 and moderate ARDS were admitted to a respiratory ICU at a university hospital and ventilated in volume-controlled mode. All patients were under deep sedoanalgesia and neuromuscular blockade. A total of 510 data points on ventilatory parameters—including plateau pressure (Pplat), driving pressure (ΔP), positive end-expiratory pressure (PEEP), tidal volume (Vt), respiratory rate (RR), and elastance—were collected from March to May 2021. MP was calculated using both the Gattinoni-S and Giosa formulas based on the collected data. Correlation analyses were performed to evaluate the relationships between MP and its individual ventilatory components, and comparisons were made between the results obtained from the different MP calculation formulas.
[18]	Determine the distributions and trajectories of ventilatory ratio and MP in patients with prolonged ventilation after COVID-19 pneumonia, comparing with a cohort of respiratory failures from other etiologies. Secondary outcomes included weaning failure rates and the ability of ventilatory ratio and MP to predict weaning success or failure.	Retrospective observational cohort study with 249 participants, divided into two groups: group 1, consisting of patients with ARDS due to COVID-19, and group 2, consisting of patients with prolonged ventilatory weaning due to ARDS from other etiologies. Cases of weaning failure and success were analyzed, with failure defined as patients who were discharged while still on MV, and success defined as those who maintained spontaneous ventilation for seven days or more.	The study included tracheostomized participants transferred from an ICU to a specialized weaning unit in Germany, diagnosed with COVID-19 between March 2020 and June 2021, who met the criteria for prolonged weaning (three weaning failures over more than seven consecutive days). It also included tracheostomized patients with respiratory failure from other etiologies unrelated to COVID-19, leading to prolonged weaning, between October 2018 and June 2021. Patients who died during weaning or had neuromuscular diseases were excluded.	All 249 patients were admitted to the pressure-controlled assisted ventilation mode. The collected ventilatory variables included FiO_2_, RR, Vt, maximum inspiratory airway pressure (P max), and PEEP. The following parameters were calculated: ∆P (defined as P max—PEEP in the pressure-controlled ventilation mode), dynamic lung-thorax compliance (Dynamic lung compliance, defined as Vt/∆P aw), ventilatory ratio, and MP using the simplified formula proposed by Becher and colleagues.
[19]	Establish the statistical hypotheses regarding 28-day mortality and the MP threshold of 17 J/min in patients with respiratory failure due to SARS-CoV-2 infection.	Analytical and observational cohort study with patients admitted to the ICU due to SARS-CoV-2 infection between March 2020 and March 2022. Data were extracted from the COVID-19 patient registry of the Intensive Care Medicine Department of a hospital. Patients were divided into two cohorts based on MP values in the first 24 h after orotracheal intubation: MP < 17 J/min and MP ≥ 17 J/min. MP measurement was performed using the simplified formula proposed by Gattinoni et al. Clinical and demographic data of the patients, treatments administered in the ICU, initial ventilatory parameters, and ventilatory parameters recorded during the clinical course were collected.	The inclusion criteria were patients over 18 years old, with a confirmed diagnosis of SARS-CoV-2 infection, requiring ICU admission, and MV with proper documentation of the variables necessary for MP calculation in the supine position, after sedation and neuromuscular blockade (when required), within the first 24 h of MV.	The study included 253 patients with respiratory failure due to COVID-19 who were admitted to the ICU of a hospital in Spain between March 2020 and March 2022. Patients were stratified into two groups based on their MP during the first 24 h of MV. Clinical and demographic variables were collected, including sex, age, comorbidities (hypertension, diabetes mellitus, obesity, and dyslipidemia), and supportive interventions such as corticosteroid therapy, high-flow nasal cannula oxygen, renal replacement therapy, and prone positioning. A Bayesian statistical analysis using a beta-binomial model was employed to evaluate the strength of evidence for associations between MP and 28-day mortality, with particular focus on the 17 J/min MP threshold.
[14]	To evaluate respiratory monitoring by MP and its relationship with mortality in patients with COVID-19–related ARDS undergoing MV and prone positioning.	Patients with COVID-19-related ARDS undergoing invasive mechanical ventilation (IMV) and prone positioning. Data on MP, ventilation, and gas exchange were collected at three time points: (i) before the first prone session, (ii) during the first prone session, and (iii) during the last prone session. The relationship between MP and ventilatory ratio with hospital mortality was analyzed. Prone positioning was applied in sessions lasting at least 16 h. MV was managed with limited Vt (4–8 mL/kg predicted body weight) and inspiratory pressures (Pplat < 30 cmH_2_O). PEEP was titrated to maintain Pplat < 30 cmH_2_O and ∆P < 15 cmH_2_O. No recruitment maneuvers were performed during prone sessions. Sessions interrupted for hemodynamic instability were not recorded, and patients with severe hemodynamic instability were excluded. All patients had continuous invasive arterial pressure monitoring and were ventilated primarily in volume-controlled mode, with continuous infusions of neuromuscular blockers during the protocol.	Patients with laboratory-confirmed SARS-CoV-2 infection [positive RT-PCR test result from nasal and pharyngeal swabs], diagnosed with moderate to severe ARDS according to the Berlin criteria, who required therapeutic intervention with MV and prone positioning.	The data were collected from the electronic medical records, including admission data (demographics, anthropometrics, and comorbidities) and clinical progression during ICU hospitalization. Ventilatory adjustment parameters were recorded (PEEP, Vt, RR, and FiO_2_), ventilatory monitoring (peak pressure, Pplat, ∆P, respiratory system compliance, and minute ventilation), and pulmonary gas exchange monitoring [partial pressure of carbon dioxide in blood (PaCO_2_), PaO_2_/FiO_2_, and RR]. Vt was reported in mL/kg of predicted body weight. ∆P was calculated as the difference between Pplat and PEEP. Static compliance was calculated as Vt/(Pplat − PEEP). RR was calculated using the formula: RR = [minute ventilation (mL/min) × PaCO_2_ (mmHg)]/(predicted body weight × 100 × 37.5). MP was expressed in J/min and calculated according to the equations of Chiumello et al.: [volume-controlled ventilation] 0.098 × RR × Vt × peak pressure − 0.5 × (Pplat − PEEP) and [pressure-controlled ventilation] 0.098 × RR × Vt × (change in airway pressure during inspiration + PEEP). Ventilatory parameters and monitoring data were collected at three time points: (i) before the first prone position session, (ii) during the first prone position session (after at least six hours), and (iii) during the last prone position session. The primary outcome was hospital mortality.
[20]	To investigate ventilatory and pulmonary hemodynamic differences between patients with ARDS due to COVID-19 and those with ARDS from other causes, based on physiological and hemodynamic parameters.	Observational case–control study involving patients with respiratory failure due to COVID-19, hospitalized in April 2020. Data on MV, arterial blood gas analysis, and laboratory tests were collected. Patients were matched 1:1 with ARDS cases due to pneumonia, according to disease severity (SAPS II—Simplified Acute Physiology Score II), PaO_2_/FiO_2_ ratio, age, sex, and body mass index (BMI). All patients were under pressure-controlled ventilation with a ∆P < 15 cmH_2_O, and the target mean arterial pressure was 60–65 mmHg. Laboratory data were obtained on the same day as pulmonary artery catheterization, and hemodynamic protocols were identical between groups.	Patients with laboratory-confirmed SARS-CoV-2 infection.	Patients were positioned in the same manner used during pulmonary pressure measurement. Three consecutive measurements with a regular curve and stable baseline were accepted, provided that the variation in cardiac output did not exceed 0.5 L/min. The following parameters were then calculated: (a) Stroke volume (cardiac output/heart rate, mL). (b) Pulmonary pulse pressure (systolic pulmonary artery pressure − diastolic pulmonary artery pressure, mmHg). (c) Transpulmonary pressure gradient (mean pulmonary artery pressure − pulmonary artery wedge pressure, mmHg). (d) Diastolic pressure gradient (diastolic pulmonary artery pressure − pulmonary artery wedge pressure, mmHg). (e) Pulmonary vascular resistance (transpulmonary pressure gradient/cardiac output, Wood units). (f) Pulmonary arterial compliance (stroke volume/pulmonary pulse pressure, mL/mmHg). (g) Time constant (pulmonary arterial compliance × pulmonary vascular resistance, s). (h) The P(v − a)CO_2_/C(a − v)O_2_ ratio (venous-to-arterial CO_2_ pressure difference/arterial-to-venous O_2_ content difference) was calculated as (PvCO_2_ [partial pressure of CO_2_ in venous blood] − PaCO_2_ [partial pressure of CO_2_ in arterial blood])/(CaO_2_ [arterial oxygen content] − CvO_2_ [venous oxygen content]), with CaO_2_ = (1.34 × SaO_2_ [arterial oxygen saturation] × Hb [hemoglobin]) + (0.003 × PaO_2_), and CvO_2_ = (1.34 × SvO_2_ [venous oxygen saturation] × Hb) + (0.003 × PvO_2_ [venous O_2_ partial pressure]). (i) Oxygen delivery (DO_2_) was obtained by cardiac output × CaO_2_, and oxygen consumption (VO_2_) by cardiac output × (CaO_2_ − CvO_2_). (j) The venous-arterial CO_2_ difference (P(v − a)CO_2_ gap) was PvCO_2_ − PaCO_2_. (k) Estimated dead space (Vd/Vt) was calculated as Vd/Vt = 1 − [(0.86 × VCO_2_est [estimated CO_2_ production])/(minute ventilation × PaCO_2_)], where VCO_2_est was derived from the sex-specific Harris–Benedict equation. (l) Minute ventilation was given by RR × expired Vt. (m) The ventilatory ratio was calculated as minute ventilation × PaCO_2_/(100 × predicted body weight × 40), with predicted body weight calculated using the ARDSNet formula, (n) Pulmonary shunt fraction (QS/QT) was (CcO_2_ [capillary oxygen content] − CaO_2_)/(CcO_2_ − CvO_2_). (o) The alveolar-arterial oxygen gradient (A–aDO_2_) was [FiO_2_ × (barometric pressure − 47) − (PaCO_2_/0.81)] − [(PaO_2_ + age/4) + 4], with the barometric pressure corresponding to the value on the day of the test. (p) MP was estimated as 0.098 × Vt × RR × (peak pressure − 0.5 × ∆P) (J/min).
[21]	Investigate whether higher levels of MP are associated with mortality in critically ill patients on MV, and whether this association is influenced by the diagnosis of COVID-19.	Retrospective analytical study with patients admitted to an ICU of a hospital in the United States between March 2020 and December 2021. MP (J/min) was calculated during the first 24 h of controlled MV using the following formula: 0.098 × RR × Vt × (PEEP + ½[ Pplat − PEEP] + [Peak Pressure − Pplat]). The average MP was considered over the first 24 h of MV. The primary outcome was all-cause mortality within 30 days of the initiation of invasive ventilation.	Adult patients with a confirmed diagnosis of COVID-19 by RT-PCR, who underwent controlled IMV for more than 24 h between March 2020 and December 2021, were selected for inclusion.	Primary analysis: The association between MP and mortality within 30 days of the initiation of invasive ventilation was evaluated through multivariable logistic regression analysis. Secondary analysis: The individual contribution of each parameter used in the calculation of MP (∆P, Vt, RR, and PEEP) was investigated through dominance analysis, as well as the association of MP with survival on day 28 and with the number of ventilator-free days after the initiation of ventilation. Additionally, a subgroup analysis was performed in patients with a PaO_2_/FiO_2_ ratio < 300 mmHg.
[22]	Investigate the influence of oxygenation indices and MP values, assessing their potential to predict mortality from COVID-19-related ARDS, compared to traditional indices.	The study used a dataset comprising information from 784 patients diagnosed with COVID-19, admitted to the 28-bed ICU of the hospital between March 2020 and November 2021. The diagnosis of COVID-19-associated ARDS (C-ARDS) was confirmed by chest CT and RT-PCR from nasopharyngeal swab samples. A total of 361 patients with typical radiological findings were included in the study; among them, 308 tested positive for RT-PCR. The diagnosis of C-ARDS was established on the first day of ICU admission, according to the Berlin criteria. Collected data included Sequential Organ Failure Assessment (SOFA) score, Acute Physiology and Chronic Health Evaluation II (APACHE II) score, Charlson comorbidity index, presence of comorbidities, age, sex, height, predicted body weight, ideal weight, BMI, pH, PaO_2_, PaCO_2_, and lactate levels.	Patients who did not receive IMV, stayed in the ICU for less than 24 h, were on MV for less than 24 h, had a history of chronic obstructive pulmonary disease or congestive heart failure, underwent tube thoracostomy, or received extracorporeal membrane oxygenation therapy, were excluded.	All patients were under orotracheal intubation, sedated, and ventilated, using either pressure-controlled ventilation or volume-controlled ventilation modes. During the initial seven-day period in the ICU, the following variables were recorded every minute and transferred to the software: peak pressure, Pplat (considered equal to peak pressure in pressure-controlled ventilation), ΔP (Pplat − PEEP in volume-controlled ventilation and pre-set inspiratory Δ pressure in pressure-controlled ventilation), PEEP, mean airway pressure [calculated by the ventilator: (pressure-controlled ventilation) = (peak pressure − PEEP) × (inspiratory time/total cycle time) + PEEP; (volume-controlled ventilation) = (peak pressure − PEEP) × 1/2 × (inspiratory time/total cycle time) + PEEP], RR, expiratory Vt (ΔV), compliance [(volume-controlled ventilation) ΔV/(Pplat − PEEP); (pressure-controlled ventilation) ΔV/(peak pressure − PEEP)], patient’s work of breathing, inspiratory-to-expiratory ratio (I:E ratio), and FiO_2_. For the volume-controlled ventilation mode, the simplified equation for volume control developed by Gattinoni et al. was applied, and for the pressure-controlled ventilation mode, the simplified equation for pressure control developed by Becher et al. was used.
[23]	Investigate the association between exposure to different levels of ΔP and MP with the mortality of patients with C-ARDS.	A retrospective observational study conducted in 22 ICUs in the Netherlands, involving COVID-19 patients who underwent IMV for the treatment of ARDS. This is a pre-planned secondary analysis of the study Practice of VENTilation in COVID−19 (PRoVENT–COVID).	Consecutive patients aged 18 years or older were eligible for participation if they were admitted to one of the participating ICUs and had received IMV for COVID-19-associated ARDS. The exclusion criteria were patients with spontaneous respiratory activity during more than half of the observations or those whose vital status was unknown on day 28.	Demographic data, information on pre-existing diseases, and home medication were collected at the beginning of the study. During the first hour of invasive ventilation and every eight hours thereafter, at fixed time points, ventilator settings and parameters were recorded until day four. Since Pplat was not recorded in the current study, all dynamic ΔP measurements were calculated as peak inspiratory pressure minus PEEP. Dynamic MP was calculated as 0.098 × RR × Vt × [Peak Pressure − (0.5 × Dynamic ΔP)]. Both variables were calculated considering only moments without evidence of spontaneous breathing. The Berlin definition for ARDS was used to classify the severity, categorizing it as mild, moderate, or severe. The primary outcome of the study was 28-day mortality. In secondary analyses, it was investigated whether the strength of the association between ventilation intensity and 28-day mortality changed over time. The effect of cumulative response was quantified, and it was examined whether the severity class of ARDS influenced the effects of ΔP and MP variables on 28-day mortality.
[24]	Investigate the impact of ventilation intensity on patient outcomes.	This is a secondary analysis of the PRoVENT-COVID study, a retrospective, multicenter observational study that included COVID-19 patients who underwent IMV, conducted during the first three months of the pandemic in 22 ICUs in the Netherlands. Demographic data, information on pre-existing diseases, and home medication were collected. On the first day of IMV, during the first hour after intubation, and subsequently every eight hours at fixed time points, ventilator settings and parameters were recorded.	Inclusion criteria were participants aged 18 years or older, mechanically ventilated due to respiratory failure caused by RT-PCR–confirmed COVID-19. Patients with incomplete MV data and those with missing 28-day follow-up were excluded.	First, it was determined whether there was evidence of spontaneous breathing. Spontaneous breathing was considered likely if: (a) the patient was in a spontaneous ventilation mode, such as pressure support ventilation; or (b) the patient was in an assisted-controlled ventilation mode with a measured (total) RR exceeding the defined RR by more than two breaths per minute. ΔP and MP were calculated only for time points where there was no evidence of spontaneous breathing. For each time point, dynamic ΔP and MP were calculated using the following formulas: ΔP = peak pressure − PEEP, and MP = 0.098 × Vt × RR × (peak pressure − 0.5 × ΔP). Kaplan–Meier curves were used to compare 28-day mortality between patients who received high and low average ΔP and MP. The cutoff point for ΔP was defined as 15 cmH_2_O, although the ideal threshold for dynamic ΔP is less clear than for static ΔP. The cutoff point for MP was 17 J/min. Two sensitivity analyses were performed. First, the models were rerun according to the degree of hypoxemia on the first day of invasive ventilation. For this, the cutoff points used in the Berlin definition for ARDS were applied: mild hypoxemia (200 < PaO_2_/FiO_2_ ≤ 300 mmHg), moderate (100 < PaO_2_/FiO_2_ ≤ 200 mmHg), and severe (PaO_2_/FiO_2_ ≤ 100 mmHg).
[25]	Identify a cutoff point for MP that best predicts mortality in the ICU and assess whether a longer duration with MP above this cutoff point is associated with higher mortality in the ICU.	Retrospective observational study conducted in a 28-bed general ICU of a tertiary university hospital between September 2015 and February 2022. Data were obtained from the clinical information system (CIS, Centricity Critical Care by General Electric) and the ETL (Extract, Transform, and Load) process, implemented with Structured Query Language (SQL) and Python (The article does not report the software version used). The CIS automatically collects data from all upstream devices every two minutes, including IMV parameters and laboratory values. Additionally, healthcare professionals record all patient-related information throughout the patient care process during ICU admission.	All patients aged 18 years or older, admitted to the ICU and requiring IMV for more than 24 h, were included in the study.	Demographic variables (age, sex, and BMI), patient type (medical or surgical), ICU admission type (emergency or scheduled), severity scores within 24 h of ICU admission, such as SOFA and APACHE II, comorbidities (hypertension, diabetes mellitus, chronic obstructive pulmonary disease, asthma, chronic kidney disease, and heart disease), days of IMV, ICU length of stay, and ICU mortality were collected. The relationship between peripheral oxygen saturation and FiO_2_ one hour after intubation was calculated as an indicator of oxygenation impairment. To calculate MP (J/min), the following formula was used: 0.098 × RR × Vt × [Peak Pressure − (∆P/2)]. MP values were calculated only when all components of the formula were available. Pplat values were obtained only when patients were in volume-controlled and pressure-controlled modes, and the inspiratory pause percentage was greater than 10% of the respiratory cycle. No manual inspiratory occlusion maneuver was performed. The primary outcome was to identify a MP threshold beyond which the likelihood of mortality in the ICU increased. The threshold was determined through a Lowess (locally weighted scatterplot smoothing) regression between the collected MP values and the mortality outcome for all patients. To evaluate the effect of MP above the cutoff point on days of IMV and ICU length of stay, Pearson correlation and simple linear regression were performed with the survivor data.

%: percentage, <: less than, ≤: less than or equal to, A–aDO_2_: alveolar–arterial oxygen gradient, APACHE II: acute physiology and chronic health evaluation II, ARDS: acute respiratory distress syndrome, ARDSNet: Acute Respiratory Distress Syndrome Network, aw: airway, BMI: body mass index, CaO_2_: arterial oxygen content, CcO_2_: pulmonary capillary oxygen content, CIS: Clinical Information System, cmH_2_O: centimeters of water pressure, CO_2_: carbon dioxide, COVID-19: coronavirus disease 2019, CT: computed tomography, CvO_2_: venous oxygen content, C-ARDS: acute respiratory distress syndrome associated with coronavirus disease 2019 (COVID-19), Δ (delta): change in, ΔP: driving pressure, ΔV (DeltaV): change in volume, DO_2_: oxygen delivery, ETL: extract, transform, and load, FiO_2_: fraction of inspired oxygen, Hb: hemoglobin, ICU: intensive care unit, I:E: inspiratory-to-expiratory ratio, IMV: invasive mechanical ventilation, J/min: joules per minute, L/min: liters per minute, LOWESS: locally weighted scatterplot smoothing, mL: milliliters, mL/min: milliliters per minute, mL/kg: milliliters per kilogram of predicted body weight, mL/mmHg: milliliters per millimeter of mercury, mmHg: millimeters of mercury, MP: mechanical power, MV: mechanical ventilation, PaCO_2_: partial pressure of carbon dioxide in arterial blood, PaO_2_: partial pressure of oxygen in arterial blood, PEEP: positive end-expiratory pressure, P max: maximum inspiratory airway pressure, pH: potential of hydrogen, Pplat: plateau pressure, PRoVENT-COVID: The PRactice of VENTilation in COVID-19 Study, PvCO_2_: partial pressure of carbon dioxide in venous blood, PvO_2_: Partial pressure of oxygen in venous blood, QS/QT: pulmonary shunt fraction, RR: respiratory rate, RT-PCR: reverse transcription polymerase chain reaction, s: seconds, SaO_2_: arterial oxygen saturation, SAPS II: simplified acute physiology score II, SARS-CoV-2: severe acute respiratory syndrome coronavirus 2, SOFA: sequential organ failure assessment, SQL: Structured Query Language, SvO_2_: venous oxygen saturation, VCO_2_: carbon dioxide production, VCO_2_est: estimated carbon dioxide production, Vd/Vt: dead space fraction, VO_2_: oxygen consumption, Vt: tidal volume, WHO: world health organization.

**Table 3:** Description of the results and conclusions of the studies included in this systematic review.

Ref	Results	Conclusion
[13]	The mean length of hospital stay was 22 days, with 64% of patients resulting in mortality. The mean positive end-expiratory pressure (PEEP) was 12.1 cmH_2_O, and the mean plateau pressure (Pplat) was 26.1 cmH_2_O. The average tidal volume (Vt) was 360 mL, with a mean respiratory rate (RR) of 32 breaths per minute. Mean mechanical power (MP) calculated using Gattinoni’s formula was 26.9 J/min, while Giosa’s formula yielded 30.3 J/min. The correlation between Gattinoni-S and Giosa was 0.98. Component correlations were as follows: elastance with driving pressure (ΔP), 0.88; elastance with PEEP, −0.54; and elastance with Vt, −0.44.	During the first 24 h of mechanical ventilation (MV), MP was not an additional predictor of mortality in coronavirus disease 2019 (COVID-19) patients. However, after 48 h, parameters related to the elastic component of ventilation became indicative of poorer prognosis, while PEEP remained an equally relevant parameter for outcome assessment.
[18]	The study included 249 of the 256 original participants; four had died and three had neuromuscular diseases. Among the 249 patients, 53 required prolonged MV following acute respiratory distress syndrome (ARDS) associated with laboratory-confirmed COVID-19 pneumonia. The mean age was 69 years, with 168 males, and a mean body mass index (BMI) of 28.4 kg/m^2^. A history of smoking was present in 89 patients, and the mean Acute Physiology and Chronic Health Evaluation II (APACHE II) score was 15. The most prevalent comorbidities were systemic arterial hypertension and diabetes mellitus. Regarding ventilatory parameters, the COVID-19 group exhibited higher RR, MP, ventilatory ratio, and Vt compared to controls during initial data collection. By the end of the study, these variables did not differ significantly between groups. The COVID-19 group had a significantly lower weaning failure rate (9% vs. 30%). Median weaning duration was similar between groups [13 (IQR = 11–19) vs. 12 (IQR = 10–17) days], but total duration of invasive mechanical ventilation (IMV) was longer in the COVID-19 group [48 (IQR = 36–64) vs. 40 (IQR = 31–53) days].	The COVID-19 patients undergoing prolonged MV exhibited higher MP during the weaning process compared to patients with respiratory failure from other causes. However, this increased mechanical load was associated with greater lung compliance, which may explain the lower weaning failure rate observed in COVID-19 patients. Differences in ventilation and respiratory mechanics were particularly evident among those requiring prolonged MV and tracheostomy, resulting in higher ventilatory ratio and MP during weaning. The observed differences in MP were linked to increased lung–chest compliance in COVID-19 patients, potentially accounting for their lower weaning failure rate. An independent association was identified between weaning failure and specific MP, but not with ventilatory ratio or overall MP.
[19]	A total of 911 patients were admitted to the intensive care unit (ICU) due to severe acute respiratory syndrome coronavirus 2 (SARS-CoV-2) infection, of whom 552 met the study inclusion criteria. Complete data were available for 253 patients, who were divided into two groups based on MP during the first 24 h of MV. Initial RR (Bayes Factor, BF_10_ = 3.83 × 10^6^), peak pressure (BF_10_ = 3.72 × 10^13^), and pneumothorax development (BF_10_ = 17,663) were the parameters most likely to differ between the groups. Bayesian binomial analysis showed that, in the group with MP < 17 J/min, BF_10_ was 12.71, indicating that the evidence supporting the alternative hypothesis was 12.71 times stronger than that for the null hypothesis. The 95% confidence interval for the proportion of patients in this group was 0.27–0.58. In the group with MP ≥ 17 J/min, BF_10_ was much higher (36,100), indicating strong evidence in favor of the alternative hypothesis, with a 95% confidence interval of 0.42–0.72 for the proportion of patients.	A MP ≥ 17 J/min was strongly associated with increased 28-day mortality, as indicated by a high BF_10_ and a very low BF_01_, providing evidence for this threshold as a predictor of poor outcome.
[14]	Data were collected from 91 patients with COVID-19-associated ARDS, all intubated and mechanically ventilated in the prone position. ICU and hospital mortality was 49% (n = 45). Most patients were male (63.7%), with a mean age of 60.2 ± 12.8 years and a median BMI of 30 kg/m^2^ (IQR = 26.8–34.6). The mean Simplified Acute Physiology Score III (SAPS III) at ICU admission was 68.6 ± 15.5, and the mean Sequential Organ Failure Assessment (SOFA) score was 7 ± 2.3. The median number of prone sessions was 2 (IQR = 1–4). During the first prone session, MP did not differ between survivors and non-survivors (24.5 J/min [22.1–28.4] vs. 25.7 J/min [21.7–30]). However, in the last prone session, survivors exhibited lower MP compared with non-survivors (26.1 J/min [21.6–30] vs. 32.8 J/min [26.1–38.2]). Non-survivors were older (65.6 ± 11.8 vs. 55.0 ± 11.6), had higher SAPS III scores (73.7 ± 17.1 vs. 63.8 ± 12.1), and a higher prevalence of chronic obstructive pulmonary disease (22.2% vs. 2.2%). Additionally, non-survivors underwent more prone sessions [3.0 (2.0–5.5) vs. 2.0 (1.0–4.0)].	The study demonstrated that MP is a relevant prognostic indicator, particularly in the later stages of prone-position therapy in patients with COVID-19–associated ARDS.
[20]	Patients with COVID-19-associated ARDS received MV due to SARS-CoV-2 infection, pneumonia, and respiratory failure. They were matched to a historical cohort of non-COVID-19 ARDS patients previously treated in the ICU. Simplified Acute Physiology Score II (SAPS II) scores were similar between groups (42 for COVID-19, n = 10; 41 for non-COVID-19 ARDS, n = 10), and ARDS severity ranged from mild to severe in both cohorts. PaO_2_/FiO_2_ (P/F, ratio between partial pressure of oxygen in arterial blood and fraction of inspired oxygen) ratios were also comparable (median 118 vs. 120). The median age was 71 years (range 45–85) in the COVID-19 group and 62 years (range 59–71) in the non-COVID-19 group, with no significant difference. Most patients in both groups were male. BMI distribution was similar, with three COVID-19 patients classified as class I obesity, three with normal BMI, and four overweight. A notable difference was observed in body temperature: COVID-19 patients exhibited fever (median 38.5 °C) compared with non-COVID-19 ARDS patients (median 37.4 °C). Although severe respiratory failure in COVID-19 resembled classical ARDS in ventilatory characteristics, hemodynamic profiles differed: COVID-19 patients had higher MP (23.4 ± 8.9 vs. 15.9 ± 4.3 J/min), lower pulmonary vascular resistance, and higher cardiac output, despite similar pulmonary arterial pressures.	While COVID-19-associated ARDS shares similar ventilatory characteristics with classical ARDS, it is distinguished by unique hemodynamic alterations. Patients with COVID-19 exhibited higher MP, lower pulmonary vascular resistance, and increased cardiac output, indicating a specific microcirculatory dysfunction. These findings suggest that, despite comparable ventilation strategies, pulmonary vascular behavior in COVID-19 differs from that in non-COVID-19 ARDS, highlighting the importance of hemodynamic monitoring and individualized management in this population.
[21]	A total of 1737 patients were included, of whom 509 (29%) died within 30 days of initiating IMV. The cohort’s median MP was 14.5 J/min (IQR = 10.7–19.8), and the median duration of ventilation was 107.2 h (IQR = 53.4–234.6). Among participants, 411 (23.7%) had COVID-19, with a longer ventilation duration compared to non-COVID-19 patients (median 247 h [IQR = 121–442] vs. 86 h [IQR = 46–173]). Mean MP was higher in COVID-19 patients (19.9 ± 7.5 J/min) than in others (14.8 ± 6.5 J/min). Patients who died within 30 days had higher MP (18.0 ± 7.8 J/min) compared with survivors (15.2 ± 6.7 J/min). In unadjusted analyses, higher MP was associated with 30-day mortality (OR = 1.45 per 1 SD increase, 7.1 J/min; 95% CI = 1.31–1.60), with no significant interaction between COVID-19 status and MP. Among ventilatory parameters, RR contributed most to 30-day mortality. Elevated MP was also associated with fewer ventilator-free days up to day 28 (adjusted IRR = 0.83 per 1 SD increase, 7.1 J/min; 95% CI = 0.75–0.91), with an adjusted absolute difference of −2.7 days (95% CI = −4.0 to −1.3). This effect was more pronounced in COVID-19 patients (−3.96 days; 95% CI = −6.19 to −1.72) compared with non-COVID-19 patients (−1.96 days; 95% CI = −3.56 to −0.36). Exploratory analyses confirmed significant associations between higher MP and ICU mortality (adjusted OR = 1.33; 95% CI = 1.14–1.55), 7-day mortality (adjusted OR = 1.23; 95% CI = 1.03–1.48), 14-day mortality (adjusted OR = 1.25; 95% CI = 1.09–1.49), and 28-day mortality (adjusted OR = 1.27; 95% CI = 1.10–1.48). None of these associations were modified by COVID-19 status.	The study concluded that higher MP during the first 24 h of ventilation was associated with increased 30-day mortality. Additionally, patients with elevated MP experienced fewer ventilator-free days up to day 28, with this effect being more pronounced in those with COVID-19. These findings highlight the prognostic importance of MP in critically ill patients receiving IMV.
[22]	The study included a cohort of 361 ICU patients with COVID-19–associated ARDS. Clinical data were continuously collected over the first seven days of ICU admission, totaling 7212 h of monitoring (approximately 60 min per data point), with simultaneous recordings of blood gas measurements and respiratory parameters. On the first day, ARDS severity according to the Berlin classification was 107 patients (29.6%) with mild, 217 (60.1%) with moderate, and 37 (10.2%) with severe ARDS. ICU mortality was higher among patients whose P/F ratios and PaO_2_/FiO_2_ × PEEP indices were below the identified cutoffs values of 172 and 16.5, respectively. Similarly, patients with MP above the identified cutoff exhibited higher ICU mortality, whereas those with MP below the cutoff had more favorable outcomes. These findings underscore the prognostic value of these parameters for risk stratification and ventilatory management in COVID-19 ARDS. Oxygenation and saturation indices based on ΔP (OI-ΔPinsp, OSI-ΔPinsp) and dynamic MP (OI-MPdyn, OSI-MPdyn) showed strong predictive performance for mortality. Critical values of these indices were associated with higher ICU mortality and longer durations of MV.	These findings suggest that integrating parameters such as ΔP and dynamic ΔP into oxygenation and saturation indices can enhance prognostic assessment and support therapeutic decision-making in critically ill patients with COVID-19-associated ARDS. By identifying patients at higher risk of ICU mortality and prolonged MV, these indices may provide valuable guidance for individualized ventilatory management.
[23]	A total of 1340 individuals were assessed. Among 1122 COVID-19 patients receiving IMV, 734 (66.6%) were included in the final analysis. Exclusion criteria were evidence of spontaneous respiratory activity in more than 50% of data points (n = 368) and missing 28-day vital status (n = 20). Median age was 65 years (IQR = 57–72), and 26% (n = 94) were female. ARDS severity was mild in 8.9%, moderate in 57.8%, and severe in 33.4%. The most prevalent comorbidities were systemic arterial hypertension and diabetes mellitus. The 28-day mortality rate was 29.2%. During the first four days of ventilation, higher temporal variability in ΔP (HR = 1.04; 95% CrI = 1.01–1.07) and MP (HR = 1.12; 95% CrI = 1.01–1.36) were associated with increased 28-day mortality. Additionally, a greater proportion of measurements with ΔP > 15 cmH_2_O (HR = 1.61; 95% CI = 1.03–2.51) or MP > 17 J/min (HR = 2.42; 95% CI = 1.44–4.07) was also linked to higher mortality risk within this period.	Cumulative exposure to elevated ΔP or MP, as well as to ΔP > 15 cmH_2_O or MP > 17 J/min during the first four days of MV, was associated with an increased 28-day mortality risk.
[24]	Of the 1102 patients initially enrolled in the PRoVENT-COVID study, 825 (74.9%) were included in the final analysis. The mean age was 65 years, with a predominance of males (72.7%). Most patients had moderate ARDS, and the most frequent comorbidities were hypertension and diabetes mellitus. Overall, 227 patients (27.5%) died within 28 days. On the first day of IMV, the median ΔP was 14.0 cmH_2_O (IQR = 12.0–16.0), and the median MP was 18.5 J/min (IQR = 15.5–22.2). A ΔP > 15 cmH_2_O was observed in 270 patients (32.7%), while MP > 17 J/min was recorded in 473 patients (57.3%). ΔP was not associated with 28-day mortality in either univariable (HR = 1.09; 95% CI = 0.96–1.24) or multivariable analysis (HR = 1.02; 95% CI = 0.88–1.18). In contrast, MP showed a significant association with mortality in both univariable (HR = 1.17; 95% = CI 1.02–1.33) and multivariable models (HR = 1.17; 95% CI = 1.01–1.36). While mortality did not differ between patients with ΔP > 15 cmH_2_O and those with ≤15 cmH_2_O, it was markedly higher among patients with MP > 17 J/min compared with those at or below this threshold. No interactions were found between ΔP or MP and baseline hypoxemia severity regarding 28-day mortality.	Higher MP was associated with increased 28-day mortality. Both ΔP and MP proved useful as prognostic biomarkers in COVID-19 patients receiving IMV. Ventilatory strategies aimed at reducing not only ΔP but also MP may help improve clinical outcomes in this population.
[25]	During the study period, 10,874 patients were admitted to the ICU, of whom 2623 required IMV for more than 24 h. Most were male (70%), with a median age of 64 years (IQR = 53–72), BMI of 26 kg/m^2^ (IQR = 24–29), SOFA score of 5 (IQR = 4–7), and APACHE II score of 21 (IQR = 15–25). One hour after intubation, the median SpO_2_ (peripheral oxygen saturation)/FiO_2_ ratio was 217 (IQR = 158–279). Clinical conditions accounted for 71% of admissions, with hypertension (30%) and diabetes mellitus (15%) as the most frequent comorbidities. The median ICU stay was 12 days (IQR = 6–24), and duration of invasive ventilation was 6 days (IQR = 3–15). During admission, 21% underwent tracheostomy and 8% required reintubation. The median MP across all controlled ventilation modes was 16 J/min (IQR = 13–21), with an overall ICU mortality of 28%. Extreme value analysis identified a critical MP threshold of 17.9–18.0 J/min, and >18 J/min was defined as the cutoff for increased mortality risk. The median cumulative exposure above this threshold was 34 h (IQR = 8–125). Time spent with MP > 18 J/min was associated with ICU mortality. In multivariable logistic regression, this variable remained an independent predictor (OR = 1.001; 95% CI = 1.0001–1.001; AUC = 0.70), indicating that each additional hour above this threshold increased the probability of death by approximately 0.1%. Of the cohort, 277 patients (11%) were admitted for SARS-CoV-2 pneumonia. Compared with non-COVID-19 patients, they showed greater impairment in protective ventilation parameters, though ICU mortality did not differ significantly (30% vs. 28%). In this subgroup, however, the impact of elevated MP was more pronounced: each additional hour with MP > 18 J/min increased the risk of ICU death by approximately 0.3%.	The number of hours with MP exceeding 18 J/min was associated with ICU mortality in critically ill patients. Continuous monitoring of MP under controlled modes, supported by automated clinical information systems, may serve as a valuable tool to provide early alerts of increased risk and enable timely therapeutic interventions.

±: plus or minus, %: percentage, 95% CI: 95% confidence interval, 95% CrI: 95% credible interval, °C: degrees Celsius, APACHE II: acute physiology and chronic health evaluation II, ARDS: acute respiratory distress syndrome, AUC: area under the curve, BF_01_: Bayes factor supporting the null hypothesis, BF_10_: Bayes factor supporting the alternative hypothesis, BMI: body mass index, cmH_2_O: centimeters of water, COVID-19: coronavirus disease 2019, ΔP: driving pressure, FiO_2_: fraction of inspired oxygen, HR: hazard ratio, ICU: intensive care unit, IMV: invasive mechanical ventilation, IQR: interquartile range, IRR: incidence rate ratio, J/min: joules per minute, kg/m^2^: kilograms per square meter, mL: milliliters, MP: mechanical power, MV: mechanical ventilation, n: number of participants or observations, OI-ΔPinsp: oxygenation index based on inspiratory driving pressure, OI-MPdyn: oxygenation index based on dynamic mechanical power, OSI-ΔPinsp: oxygenation saturation index based on inspiratory driving pressure, OSI-MPdyn: oxygenation saturation index based on dynamic mechanical power, OR: odds ratio, PaO_2_: partial pressure of oxygen in arterial blood, PEEP: positive end-expiratory pressure, P/F: PaO_2_/FiO_2_ ratio, Pplat: plateau pressure, PRoVENT-COVID: The PRactice of VENTilation in COVID-19 Study, RR: respiratory rate, SARS-CoV-2: severe acute respiratory syndrome coronavirus 2, SAPS II: simplified acute physiology score II, SAPS III: simplified acute physiology score III, SD: standard deviation, SOFA: sequential organ failure assessment, SpO_2_: peripheral oxygen saturation, Vt: tidal volume, vs.: versus.

**Table 4:** Mathematical equations used to calculate mechanical power (MP) and the variables required for their application.

Study (Reference)	Mechanical Power Equation(s)
Influence of mechanical power and its components on mechanical ventilation in SARS-CoV-2 [13]	Equation (1). Gattinoni-S: MP = 0.098 × F × Vt × [Ppeak − 0.5 × (Pplat − PEEP)] Equation (2). Giosa: MP = 0.098 × VE × (Ppeak + PEEP + F/6)/20
Ventilatory ratio and mechanical power in prolonged mechanically ventilated COVID-19 patients versus respiratory failures of other etiologies [18]	Pressure-controlled ventilation (slope): MP = 0.098 × RR × [(ΔPinsp + PEEP) × Vt − ΔPinsp^2^ × C × (0.5 − R·C/Tslope + (R·C/Tslope)^2^ × (1 − e−Tslope/(R·C)))]
The Bayes factor in the analysis of mechanical power in patients with severe respiratory failure due to SARS-CoV-2 [19]	MP = RR × {ΔV^2^ × [0.5 × ELrs + RR × ((1 + I:E)/(60 × I:E)) × Raw] + ΔV × PEEP}
Mechanical power in prone position intubated patients with COVID-19-related ARDS: A cohort study [14]	Volume-controlled ventilation: MP = 0.098 × RR × Vt × [Ppeak − 0.5 × (Pplat − PEEP)] Pressure-controlled ventilation: MP = 0.098 × RR × Vt × (ΔPinsp + PEEP)
Pulmonary hemodynamics and ventilation in patients with COVID-19-related respiratory failure and ARDS [20]	MP = 0.098 × Vt × RR × (Ppeak − 0.5 × DP)
Mechanical power and 30-day mortality in mechanically ventilated, critically ill patients with and without coronavirus disease-2019: A hospital registry study [21]	MP = 0.098 × RR × Vt × [PEEP + 0.5 × (Pplat − PEEP) + (Ppeak − Pplat)]
Novel oxygenation and saturation indices for mortality prediction in COVID-19 ARDS patients: The impact of driving pressure and mechanical power [22]	Total MP = volume-controlled ventilation = 0.098 × RR × ΔV × (Ppeak − DP/2) Total MP = pressure-controlled ventilation = 0.098 × RR × ΔV × (PEEP + ΔPinsp) Dynamic MP = pressure-controlled ventilation = 0.098 × RR × ΔV × ΔPinsp Dynamic MP = volume-controlled ventilation = 0.098 × RR × ΔV × (Ppeak − DP/2 − PEEP)
Association of time-varying intensity of ventilation with mortality in patients with COVID-19 ARDS: Secondary analysis of the PRoVENT-COVID study [23]	Dynamic MP = 0.098 × RR × Vt × [Ppeak − 0.5 × dynamic ΔP]
Association of intensity of ventilation with 28-day mortality in COVID-19 patients with acute respiratory failure: insights from the PRoVENT-COVID study [24]	MP = 0.098 × Vt × RR × (Ppeak − 0.5 × ΔP)
Impact of mechanical power on ICU mortality in ventilated critically ill patients: A retrospective study with continuous real-life data [18]	MP = 0.098 × RR × Vt × [Ppeak − (DP/2)]

ΔPinsp: inspiratory pressure above PEEP, ΔV: volume change (used as tidal volume in the corresponding equation), ARDS: acute respiratory distress syndrome, C: respiratory system compliance, COVID-19: coronavirus disease 2019, DP: driving pressure, ELrs: respiratory system elastance, F: respiratory frequency, ICU: intensive care unit, I:E: inspiratory-to-expiratory ratio, MP: mechanical power, PEEP: positive end-expiratory pressure, Ppeak: peak inspiratory pressure, Pplat: plateau pressure, PRoVENT-COVID: The PRactice of VENTilation in COVID-19 Study, RR: respiratory rate, Raw: airway resistance, R·C: respiratory system time constant (airway resistance × respiratory system compliance), SARS-CoV-2: severe acute respiratory syndrome coronavirus 2, Tslope: inspiratory pressure rise time, VE: minute ventilation, Vt: tidal volume.

## Data Availability

No new data were created or analyzed in this study. Data sharing is not applicable to this article.

## Supplementary Materials

The following supporting information can be downloaded at: https://www.mdpi.com/article/10.3390/jcm15166476/s1, Table S1: PRISMA 2020 Checklist.

## Abbreviations

The following abbreviations are used in this manuscript:

%	Percentage
<	Less than
≤	Less than or equal to
°C	Degrees Celsius
A–aDO_2_	Alveolar–arterial oxygen gradient
95% CI	95% confidence interval
95% CrI	95% credible interval
APACHE II	Acute physiology and chronic health evaluation II
ARDS	Acute respiratory distress syndrome
ARDSNet	Acute Respiratory Distress Syndrome Network
AUC	Area under curve
aw	Airway
BF_01_	Bayes factor in favor of the null hypothesis
BF_10_	Bayes factor in favor of the alternative hypothesis
BMI	Body mass index
C	Control
C	Respiratory system compliance
CaO_2_	Arterial oxygen content
C-ARDS	COVID-19-associated acute respiratory distress syndrome
CIS	Clinical information system
CIBERES	Centro de Investigación Biomédica en Red de Enfermedades Respiratorias
cmH_2_O	Centimeters of water pressure
CO_2_	Carbon dioxide
COVID-19	Coronavirus disease 2019
CT	Computed tomography
CvO_2_	Venous oxygen content
DO_2_	Oxygen delivery
DP	Driving pressure
Δ	Change in
ΔP	Driving pressure
ΔPinsp	Inspiratory pressure above PEEP
ΔV	Change in volume
ELrs	Respiratory system elastance
ETL	Extract, transform, and load
F	Respiratory frequency
FiO_2_	Fraction of inspired oxygen
Hb	Hemoglobin
HR	Hazard ratio
I	Intervention
ICU	Intensive care unit
I:E	Inspiratory-to-expiratory ratio
IMV	Invasive mechanical ventilation
IQR	Interquartile range
IRR	Incidence rate ratio
J/min	Joules per minute
kg/m^2^	Kilograms per square meter
L/min	Liters per minute
LOWESS	Locally weighted scatterplot smoothing
mL	Milliliters
mL/kg	Milliliters per kilogram
mL/min	Milliliters per minute
mL/mmHg	Milliliters per millimeter of mercury
mmHg	Millimeters of mercury
MP	Mechanical power
MV	Mechanical ventilation
O	Outcome
OI-ΔPinsp	Oxygenation index based on inspiratory driving pressure
OSI-ΔPinsp	Oxygenation saturation index based on inspiratory driving pressure
OR	Odds ratio
OI-MPdyn	Oxygenation index based on dynamic mechanical power
OSI-MPdyn	Oxygenation saturation index based on dynamic mechanical power
P	Population
P max	Maximum inspiratory airway pressure
PACOVID	Pulmonary hemodynamics and ventilation in patients with COVID-19-related respiratory failure and ARDS study group
PaCO_2_	Partial pressure of carbon dioxide in arterial blood
PaO_2_	Partial pressure of oxygen in arterial blood
PEEP	Positive end-expiratory pressure
pH	Potential of hydrogen
P/F	PaO_2_/FiO_2_ ratio
Ppeak	Peak inspiratory pressure
Pplat	Plateau pressure
PRoVENT-COVID	The PRactice of VENTilation in COVID-19 study
PvCO_2_	Partial pressure of carbon dioxide in venous blood
PvO_2_	Partial pressure of oxygen in venous blood
Pubmed-MEDLINE	Pubmed-Medical Literature Analysis and Retrieval System Online
QS/QT	Pulmonary shunt fraction
Raw	Airway resistance
R·C	Respiratory system time constant
RR	Respiratory rate
RT-PCR	Reverse transcription polymerase chain reaction
s	Seconds
SaO_2_	Arterial oxygen saturation
SAPS II	Simplified Acute Physiology Score II
SAPS III	Simplified Acute Physiology Score III
SARS-CoV-2	Severe acute respiratory syndrome coronavirus 2
SD	Standard deviation
SOFA	Sequential organ failure assessment
SpO_2_	Peripheral oxygen saturation
SQL	Structured query language
SvO_2_	Venous oxygen saturation
T	Time
Tslope	Inspiratory pressure rise time
UCI	Unidad de Cuidados Intensivos
VCO_2_	Carbon dioxide production
VCO_2_est	Estimated carbon dioxide production
Vd/Vt	Dead space fraction
VE	Minute ventilation
VILI	Ventilator-induced lung injury
VO_2_	Oxygen consumption
vs.	Versus
Vt	Tidal volume
WHO	World Health Organization

## Appendix A

The detailed PubMed/MEDLINE search strategy conducted by the researchers is presented in Appendix A.

(“mechanical”[All Fields] OR “mechanically”[All Fields] OR “mechanicals”[All Fields] OR “mechanics”[MeSH Terms] OR “mechanics”[All Fields] OR “mechanic”[All Fields]) AND (“power, psychological”[MeSH Terms] OR (“power”[All Fields] AND “psychological”[All Fields]) OR “psychological power”[All Fields] OR “power”[All Fields] OR “powered”[All Fields] OR “powers”[All Fields] OR “powering”[All Fields]) AND (“COVID-19”[All Fields] OR “COVID-19”[All Fields] OR “COVID-19”[MeSH Terms] OR “COVID-19 vaccines”[All Fields] OR “COVID-19 vaccines”[MeSH Terms] OR “COVID-19 serotherapy”[All Fields] OR “COVID-19 serotherapy”[MeSH Terms] OR “COVID-19 nucleic acid testing”[All Fields] OR “COVID-19 nucleic acid testing”[MeSH Terms] OR “COVID-19 serological testing”[All Fields] OR “COVID-19 serological testing”[MeSH Terms] OR “COVID-19 testing”[All Fields] OR “COVID-19 testing”[MeSH Terms] OR “SARS-CoV-2”[All Fields] OR “SARS-CoV-2”[All Fields] OR “SARS-CoV-2”[All Fields] OR “SARS-CoV-2”[All Fields] OR “SARS-CoV-2”[MeSH Terms] OR “severe acute respiratory syndrome coronavirus 2”[All Fields] OR “2019 ncov”[All Fields] OR ((“coronavirus”[MeSH Terms] OR “coronavirus”[All Fields] OR “cov”[All Fields] OR “ncov”[All Fields]) AND 2019/11/01:3000/12/31[Date —Publication]) OR (“COVID-19”[All Fields] OR “COVID-19”[All Fields] OR “COVID-19”[MeSH Terms] OR “COVID-19 vaccines”[All Fields] OR “COVID-19 vaccines”[MeSH Terms] OR “COVID-19 serotherapy”[All Fields] OR “COVID-19 serotherapy”[MeSH Terms] OR “COVID-19 nucleic acid testing”[All Fields] OR “COVID-19 nucleic acid testing”[MeSH Terms] OR “COVID-19 serological testing”[All Fields] OR “COVID-19 serological testing”[MeSH Terms] OR “COVID-19 testing”[All Fields] OR “COVID-19 testing”[MeSH Terms] OR “SARS-CoV-2”[All Fields] OR “SARS-CoV-2”[All Fields] OR “SARS-CoV-2”[All Fields] OR “SARS-CoV-2”[All Fields] OR “SARS-CoV-2”[MeSH Terms] OR “severe acute respiratory syndrome coronavirus 2”[All Fields] OR “2019 ncov”[All Fields] OR ((“coronavirus”[MeSH Terms] OR “coronavirus”[All Fields] OR “cov”[All Fields] OR “ncov”[All Fields]) AND 2019/11/01:3000/12/31[Date—Publication])) OR (“coronavirus”[MeSH Terms] OR “coronavirus”[All Fields] OR “coronaviruses”[All Fields]) OR (“SARS-CoV-2”[MeSH Terms] OR “SARS-CoV-2”[All Fields] OR “SARS-CoV-2”[All Fields]) OR (“severe acute respiratory syndrome related coronavirus”[MeSH Terms] OR (“severe”[All Fields] AND “acute”[All Fields] AND “respiratory”[All Fields] AND “syndrome related”[All Fields] AND “coronavirus”[All Fields]) OR “severe acute respiratory syndrome related coronavirus”[All Fields] OR (“sars”[All Fields] AND “cov”[All Fields]) OR “SARS-CoV”[All Fields])).
